# DNA methylation patterns vary in boar sperm cells with different levels of DNA fragmentation

**DOI:** 10.1186/s12864-019-6307-8

**Published:** 2019-11-27

**Authors:** Abdolrahman Khezri, Birgitte Narud, Else-Berit Stenseth, Anders Johannisson, Frøydis Deinboll Myromslien, Ann Helen Gaustad, Robert C. Wilson, Robert Lyle, Jane M. Morrell, Elisabeth Kommisrud, Rafi Ahmad

**Affiliations:** 1 Department of Biotechnology, Inland Norway University of Applied Sciences, Hamar, Norway; 2 Department of Clinical Sciences, Swedish University of Agricultural Sciences, Uppsala, Sweden; 3 Topigs Norsvin, Hamar, Norway; 4 Department of Medical Genetics and Norwegian Sequencing Centre, Oslo University Hospital and the University of Oslo, Oslo, Norway

**Keywords:** Boar, Sperm, DNA-methylation, DNA-integrity, Epigenetics, RRBS

## Abstract

**Background:**

Sperm DNA integrity is considered essential for successful transmission of the paternal genome, fertilization and normal embryo development. DNA fragmentation index (DFI, %) has become a key parameter in the swine artificial insemination industry to assess sperm DNA integrity. Recently, in some elite Norwegian Landrace boars (boars with excellent field fertility records), a higher level of sperm DFI has been observed. In order to obtain a better understanding of this, and to study the complexity of sperm DNA integrity, liquid preserved semen samples from elite boars with contrasting DFI levels were examined for protamine deficiency, thiol profile and disulphide bonds. Additionally, the DNA methylation profiles of the samples were determined by reduced representation bisulphite sequencing (RRBS).

**Results:**

In this study, different traits related to sperm DNA integrity were investigated (*n* = 18 ejaculates). Upon liquid storage, the levels of total thiols and disulphide bonds decreased significantly, while the DFI and protamine deficiency level increased significantly. The RRBS results revealed similar global patterns of low methylation from semen samples with different levels of DFI (low, medium and high). Differential methylation analyses indicated that the number of differentially methylated cytosines (DMCs) increased in the low-high compared to the low-medium and the medium-high DFI groups. Annotating the DMCs with gene and CpG features revealed clear differences between DFI groups. In addition, the number of annotated transcription starting sites (TSS) and associated pathways in the low-high comparison was greater than the other two groups. Pathway analysis showed that genes (based on the closest TSS to DMCs) corresponding to low-high DFI comparison were associated with important processes such as membrane function, metabolic cascade and antioxidant defence system.

**Conclusion:**

To our knowledge, this is the first study evaluating DNA methylation in boar sperm cells with different levels of DFI. The present study shows that sperm cells with varying levels of DNA fragmentation exhibit similar global methylation, but different site-specific DNA methylation signatures. Moreover, with increasing DNA fragmentation in spermatozoa, there is an increase in the number of potentially affected downstream genes and their respective regulatory pathways.

## Background

Sperm cells have a different chromatin structure compared to somatic cells. In somatic cells, DNA is wrapped around histone proteins, which allows DNA condensation. In contrast, during spermatogenesis histone proteins are, to a great extent, replaced by protamines coupled by disulphide bridges, a process that facilitates tight packaging of DNA in the sperm nucleus [1]. Sperm cells are responsible for transmitting the paternal genetic material to the oocyte and contributing to the development of a viable embryo. Therefore, the integrity of sperm chromatin is crucial. A wide range of internal and external factors such as abnormal spermatid maturation, abortive apoptosis of germ cells, oxidative stress, semen handling methods, environmental stressors, age and bacterial infections can result in sperm DNA fragmentation [2].

Epigenetics is a phenomenon where a series of events such as DNA methylation, histone post-translational modification (PTM) and close association with small RNAs, independently or in concert, control gene expression, without altering the DNA sequence [3]. During spermatogenesis, sperm cells undergo a high level of epigenetic reprogramming, reflected by histone PTM and sperm DNA methylation, which is initiated by the erasure of DNA methylation in the primordial germ cells followed by de novo DNA methylation [1]. In developing germ cells, DNA methylation occurs in specific DNA regions by adding a methyl group to the 5th carbon of cytosine (C) in CpG dinucleotides [4]. It has been shown that DNA methylation is dynamic and might be affected by a wide range of environmental stress factors [3].

Both sperm DNA methylation and fragmentation have been reported to correlate with fertility and field performance in different livestock. For instance, it has been shown that site-specific sperm DNA methylation status correlates with infertility in boars [5] and reproductive efficiency in bulls [6]. In addition, previous research has documented that sperm DNA fragmentation is significantly correlated with field fertility performance in boars [7–9] and aberrant embryo development in mammals [10, 11].

Reduced representation bisulphite sequencing (RRBS) allowing the study of methylation profiles at single-base resolution, while experiment costs are kept low [12]. RRBS is an efficient and high-throughput method and previous studies have used RRBS to investigate DNA methylation profiles in different tissues in pigs [13, 14]. However, the RRBS has not previously been employed to investigate DNA methylation in boar sperm.

The liquid diluted boar semen produced for pig production in Norway is recommended to be used within 96 h upon collection. However, due to factors such as long-distance transport and a long shipment time, the semen is often stored for 48 to 96 h prior to artificial insemination (AI) [7]. Recently, it has been reported that sperm DNA fragmentation in Norwegian Landrace show a small, but significant increase in DFI upon 96 h liquid storage [7]. In addition, it has been recently reported that 1.7% of ejaculates from elite Norwegian Landrace boars with a well-known pedigree, have DNA fragmentation index (DFI, %) values above 10% [7]. Therefore, it became of particular interest to analyse other parameters related to chromatin integrity (thiol profile, disulphide bonds, protamine deficiency) and DNA methylation in sperm cells. The aim of this study was to investigate the differences in the above-mentioned chromatin integrity parameters upon storage and to use RRBS for evaluation of DNA methylation in liquid stored ejaculates with different levels of DFI.

## Results

### Phenotypic assessment of boar sperm cells

An overview of sperm DNA integrity parameters is presented in Table 1. Sperm cells from Day 4 showed a significant reduction in total thiols and disulphide bonds and a significant increase in protamine deficiency level compared to Day 0 semen samples. The level of free thiols was the only sperm parameter that showed no significant change between Day 0 and Day 4 semen samples. Moreover, the results indicate that DFI was the most contrasting DNA integrity parameter with higher levels at Day 4 compared to Day 0 among individuals (Table 1). This is supported by a 67-fold difference between the maximum and minimum DFI values in individuals both at Days 0 and 4. Therefore, samples were categorized as low (L), medium (M) and high (H) groups based on their DFI value for downstream RRBS analysis.

**Table 1:** Assessment of phenotypic traits related to boar sperm DNA integrity. Data (*n* = 18) related to sperm DNA integrity parameters on the day of semen collection (Day 0) and upon liquid preservation at 18 °C for 96–108 h (Day 4), shown as mean ± SEM. For DFI at Day 0 (*n* = 13)

		Day 0	Day 4
Free thiols (mFI)	min – max	4848.0–12,295.3	4401.3–13,353.0
	mean ± SEM	7133.7 ± 557.8	7489.5 ± 657.2
Total thiols (mFI)	min – max	42,778.6–48,306.3	33,912.7–45,298.0
	mean ± SEM	44,850.7 ± 374.3	41,477.0 ± 705.1 ***
Disulphide bonds (mFI)	min – max	16,841.8–21,729.1	11,656.3–20,143.1
	mean ± SEM	18,854.5 ± 263.6	16,993.7 ± 501.7***
Protamine deficiency (mFI)	min – max	2386.1–3719.4	2608.5–4756.0
	mean ± SEM	2915.9 ± 94.8	3553 ± 164.4 ***
DFI (%)	min – max	0.3–20.4	0.4–27.4
	mean ± SEM	6.0 ± 1.6	7.8 ± 1.9 ***

Asterisks indicate a significant difference between Day 0 and Day 4 based on linear mixed model. *** indicate *p* < 0.0003 for all parameters except DFI and protamine deficiency, where *** indicate *p* < 0.001. mFI; mean fluorescence intensity, DFI; DNA fragmentation index, SEM; standard error of mean

Furthermore, potential correlation between DFI and other sperm DNA integrity parameters was investigated (Table 2). Although all parameters showed positive correlation with DFI, only free thiols and disulphide bonds exhibited a significant, albeit weak, correlation.

**Table 2:** Regression analysis between DFI and other DNA integrity parameters. Data (n = 18) related to boar sperm DNA integrity parameters and DFI, at the day of semen collection (Day 0) and upon liquid preservation at 18 °C for 96–108 h (Day 4) were merged together for correlation analysis

	DFI vs. Free thiols	DFI vs. Total thiols	DFI vs. Disulphide bonds	DFI vs. Protamine deficiency
Multiple R^2^	0.1609	0.0172	0.1295	0.0270
*p*-value	0.0228 *	0.4735	0.0430 *	0.3685

* indicates significant correlation *p* < 0.05, using multiple linear regression model. DFI; DNA fragmentation index

### Assessment of RRBS data

An overview of the RRBS libraries and their basic statistics is provided in Table 3. Briefly, the data show a successful and very consistent conversion rate (average 99.8%) of unmethylated cytosines to uracil. There was an average of 15.6 million reads per sample, 19.4x read coverage and 58.3% unique mapping efficiency, as determined using an in-house bioinformatics pipeline.

**Table 3:** An overview of basic RRBS statistics. Boar ejaculates (n = 18) from seven different individuals (boar A – G) after Day 4 of liquid preservation at 18 °C, based on their DFI value were divided into low (L), medium (M) and high (H) groups (six ejaculates each)

DFI group	DFI (%)	Sample ID	Boar ID	Total reads	Clean reads after trimming	Read coverage (X)	Mapping efficiency (%)	CpG methylation (%)	Number of CpGs (10X)	Bisulphite conversion rate (%)
**Low** **(L)**	0.4	L 1	A	16,442,959	16,372,832	20.5	60.5	12.4	1,136,877	99.9
	0.5	L 2	A	19,490,143	19,164,300	20.1	46.7	42.2	797,270	98.9
	0.6	L 3	A	21,720,429	21,600,109	27.3	57.0	41.5	1,261,660	98.9
	0.6	L 4	A	13,374,736	13,313,871	16.7	58.8	9.8	971,589	99.9
	0.7	L 5	B	12,614,151	12,582,707	15.7	63.3	42.8	438,273	99.9
	0.9	L 6	B	16,423,268	16,286,276	20.5	57.3	38.8	951,776	99.9
**Medium** **(M)**	5.8	M 1	C	13,193,208	13,168,643	16.5	62.6	44.1	475,564	99.9
	6.9	M 2	D	12,551,543	12,528,289	15.7	62.4	39.1	453,421	99.9
	6.9	M 3	C	10,703,251	10,679,744	13.4	64.2	47.0	287,489	99.9
	7.0	M 4	C	15,460,504	15,025,860	18.9	50.1	40.4	769,498	99.9
	7.5	M 5	D	14,215,843	14,142,428	17.7	56.5	12.1	975,707	99.9
	8.1	M 6	E	15,162,760	15,122,630	18.7	62.0	41.1	545,567	99.9
**High** **(H)**	15.8	H 1	F	17,995,363	17,939,071	22.6	62.2	13.1	1,183,512	99.9
	18.5	H 2	D	17,139,869	16,928,292	21.3	45.6	38.3	725,755	99.9
	21.3	H 3	F	16,767,296	16,670,788	20.8	61.2	7.9	1,145,606	99.8
	26.0	H 4	G	11,580,556	11,547,471	14.2	63.6	48.2	152,219	99.9
	27.4	H 5	F	21,075,288	20,855,183	26.3	50.7	30.8	1,188,854	99.9
	28.4	H 6	G	17,655,261	17,614,978	21.8	64.3	50.1	496,490	99.9

Clean reads were obtained after adapter and low-quality trimming of Illumina sequencing reads (total reads). Read coverage was calculated by dividing the clean reads with the in silico MspI-digested *Sus scrofa* genome. Mapping efficiency indicates the percentage of uniquely mapped clean reads with the *S. scrofa* 11.1, reference genome. CpG methylation shows the percentage of global methylation in clean reads. Downstream analyses were performed based on number of 10x CpGs. Bisulphite conversion rate shows the proportion of Cs deaminated to uracil

**Fig. 1:**
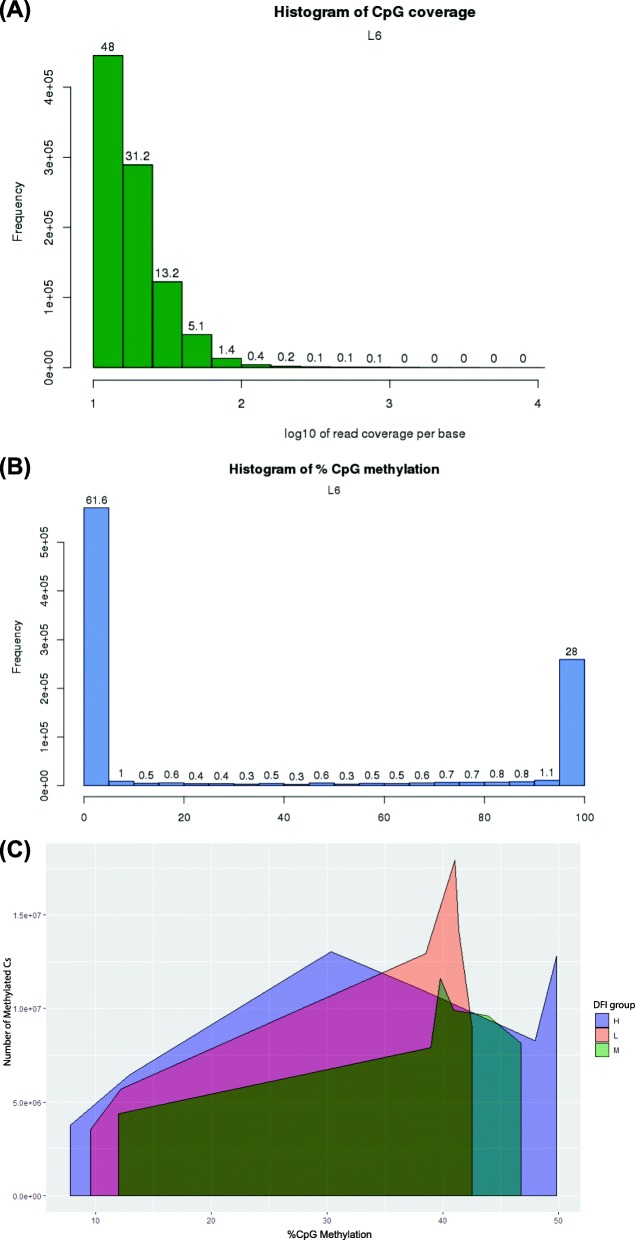
An Overview of CpG coverage and %CpG methylation**.** A) CpG site coverage histogram for one representative sample in high (H) DFI group (sample L1), where the x-axis indicates log10 values corresponding to the number of reads per CpG and y-axis denotes the number of reads. B) CpG methylation distribution for sample L1, where the x-axis indicates percent methylation at each cytosine site and y-axis indicates the number of CpGs. For both A and B, the numbers on the bars indicate the percentage in each respective bin. C) Change in %CpG methylation of methylated cytosines for all samples, in the L: low, M: medium and H: high DFI groups

CpG coverage and methylation levels for a representative sample are presented in Fig. 1 (corresponding data for all samples are available in Additional File 1). The results show that the generated libraries contained a considerable number of reads with high coverage (>10x) of the CpGs. In addition, a single peak on the left-hand side of the histogram (Fig. 1a) was observed for all the samples, which indicates that there were no overrepresented read counts and potentially minimal redundant fragment amplification in the PCR step. Distribution analysis of methylation at each CpG site showed low methylation levels (i.e., percentage methylation < 20%) for 64–94% of the CpGs (Fig. 1b and Additional File 1). Based on the overlapping density plot for the L, M and H groups (Fig. 1c), it is interesting to note a consistent shift in the %CpG methylation (H > M > L). However, the multiple regression model showed no significant correlation between DFI and the percentage of global methylation in the CpG context (multiple R^2^: 0.0046, *p*-value: 0.7877).

**Fig. 2:**
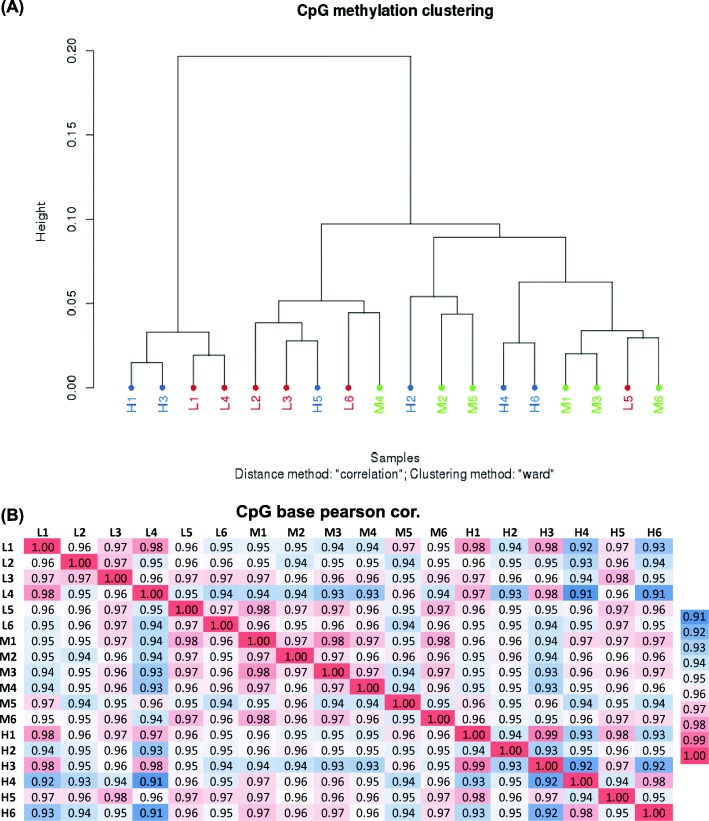
Clustering and correlation of analysis of samples based on CpG_10_ methylation level. A) Hierarchical clustering by methylation levels of CpG_10_ in different boar sperm samples with different levels of DFI. B) Heat map and correlation analysis based on CpG_10_ data among boars with different levels of DFI. Numbers in each cell represent the pairwise Pearson’s correlation scores

Cluster analysis, based on CpG_10_ (i.e., CpGs ≥10x read coverage) methylation levels, the samples are distributed in two clusters small (4 samples) and large (14 samples). However, the samples from different DFI groups don’t appear to cluster together (Fig. 2a). Also, a heat map of DNA methylation based on the same criteria (Fig. 2b), indicated a very high positive correlation between the samples (Pearson’s correlation coefficient ≥ 0.92).

### Differential methylation analysis

**Fig. 3:**
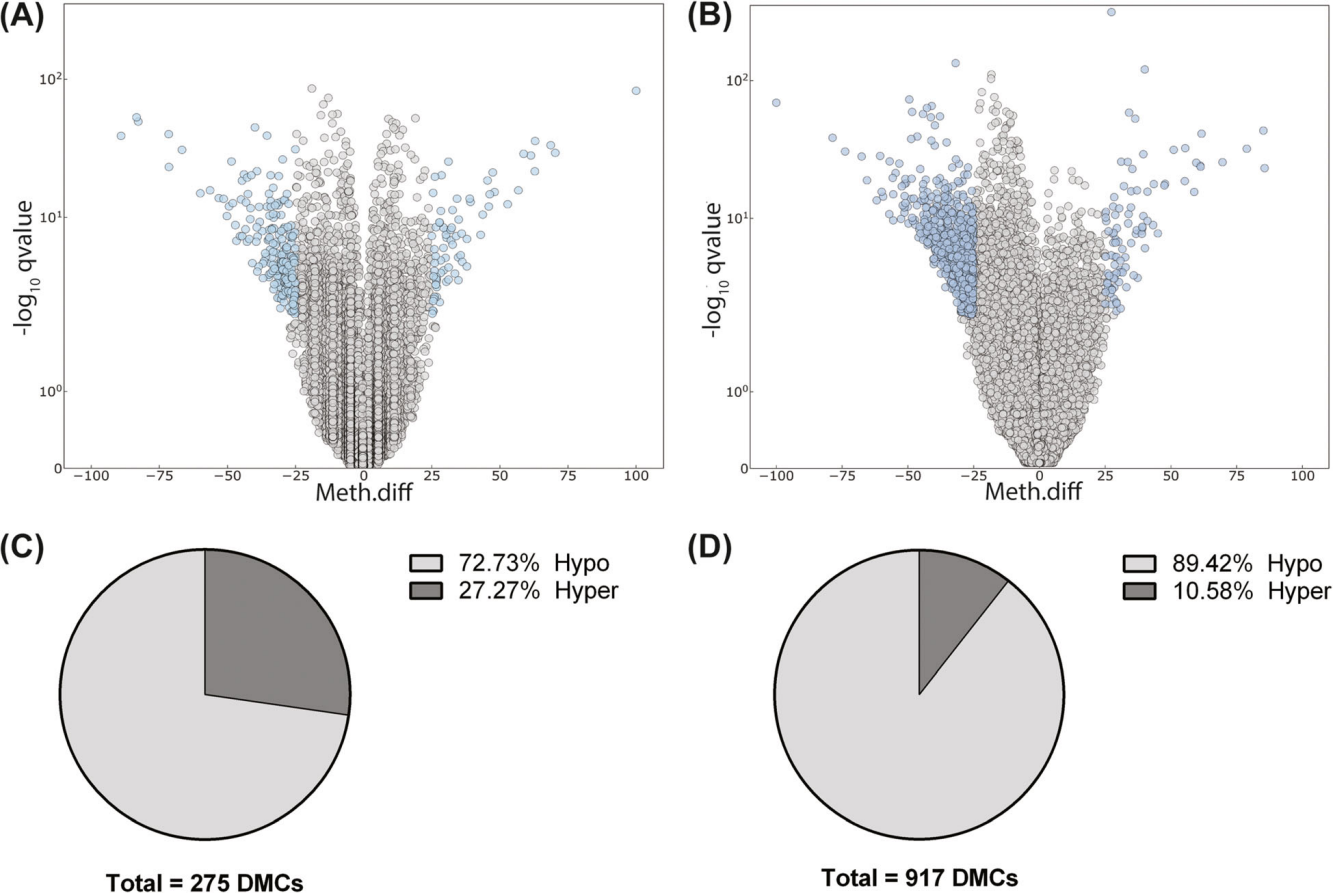
Differential methylation analysis for CpG_10_ from boars with different levels of sperm DFI. A and B: Each dot in the volcano plot represents one differentially methylated cytosine (DMC). All identified DMCs between LM (A) and LH (B) groups are plotted based on the level of methylation (x-axis) and their corresponding -log10 q-values (y-axis). Blue dots represent DMCs with over 25% methylation difference and q-value < 0.01 (filtered DMCs). C and D: Pie chart of filtered DMCs between LM (C) and LH (D) groups. LM: low – medium, LH: low – high, Hyper: hypermethylated cytosines, Hypo: hypomethylated cytosines

Filtering the reads to remove Cs exhibiting ≤10x coverage yielded 135,295 and 221,282 differentially methylated cytosines (DMCs) with varying levels of methylation ranging from 0 to 100% in the low-medium (LM) and low-high (LH) groups, respectively (Fig. 3a and b). However, after using the default differential methylation settings (cut-off 25% and q-value < 0.01), 275 and 917 DMCs were filtered out in the LM and LH groups, respectively (Fig. 3c and d). A large majority of these were found to be hypomethylated relative to the low DFI group. Interestingly, with an increase in the DFI level, both the number of DMCs and the percentage of hypomethylated Cs increased. In addition, 209 DMCs were identified in the medium-high (MH) group. Similar to the LM and LH groups, a majority of the DMCs in the MH comparison were also hypomethylated relative to the medium DFI group (Additional File 2: Fig. S1).

### Annotation of DMCs with gene and CpG features

**Fig. 4:**
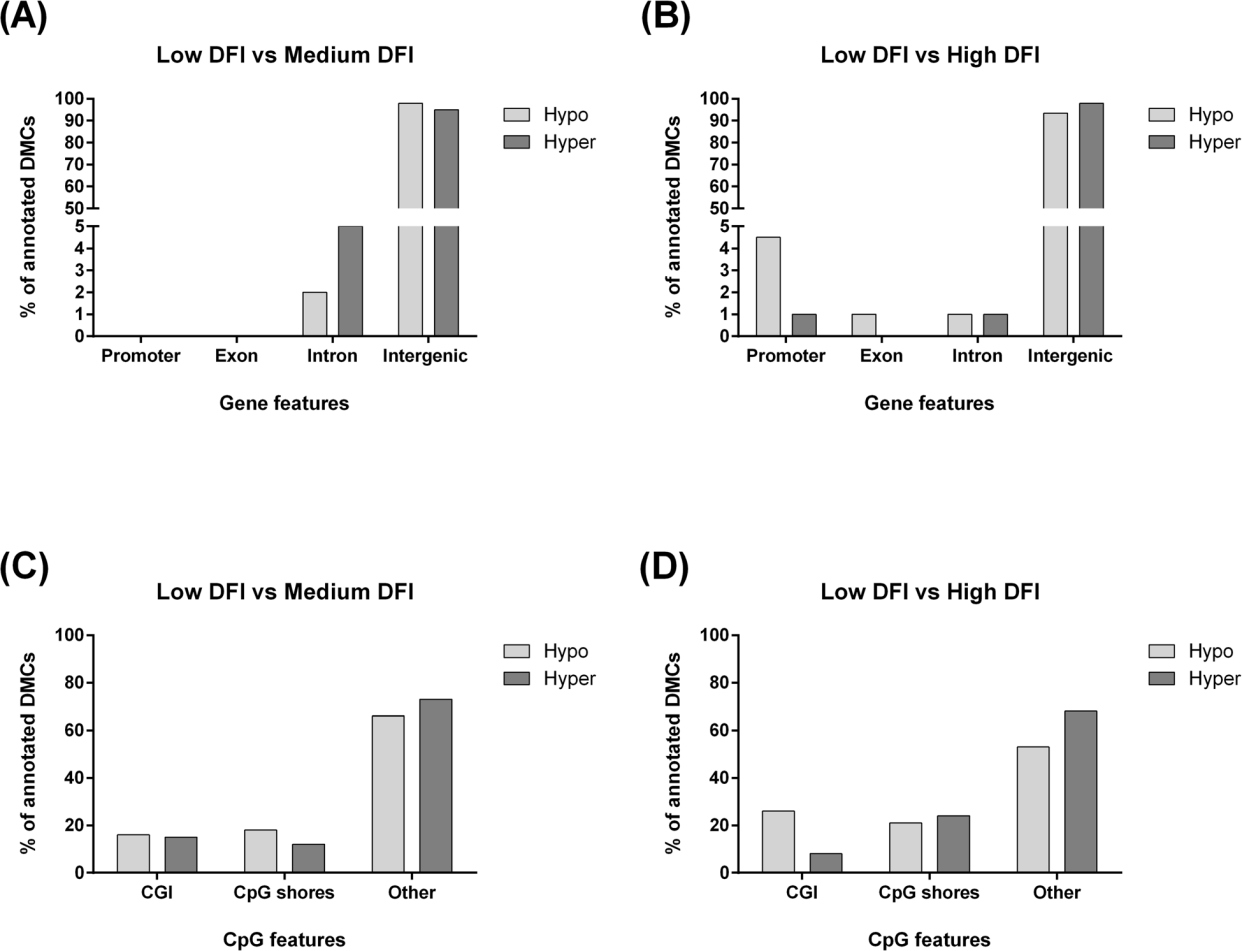
An overview of the spread of filtered DMCs in the *Sus scrofa* genome. Annotation of the filtered DMCs with gene features in A) LM, B) LH groups and with CpG features in C) LM, D) LH groups. Hyper: hypermethylated cytosines, Hypo: hypomethylated cytosines, CGI: CpG island

After differential methylation analysis, the filtered DMCs were annotated with gene and CpG features. The analysis revealed that over 90% of the filtered DMCs were present in the intergenic regions. Furthermore, none of the filtered DMCs in the LM comparison was annotated within promoters and exons, while in the LH group, 6% of filtered DMCs were annotated within these features and the majority of these were hypomethylated (Fig. 4a and b). For CpG features, 10–25% and 20–30% of filtered DMCs were annotated within CpG islands (CGI) and CpG shores, respectively, and over 55% of filtered DMCs were annotated outside of these regions (Fig. 4c and d). Interestingly, in the LH group, a 16% difference between the annotation of hypo- and hypermethylated Cs within CGI was observed (Fig. 4c and d). Also, in the MH comparison the majority of the filtered DMCs were annotated within the intergenic region and were present outside CGI and CpG shores (Additional File 2: Fig. S2).

**Fig. 5:**
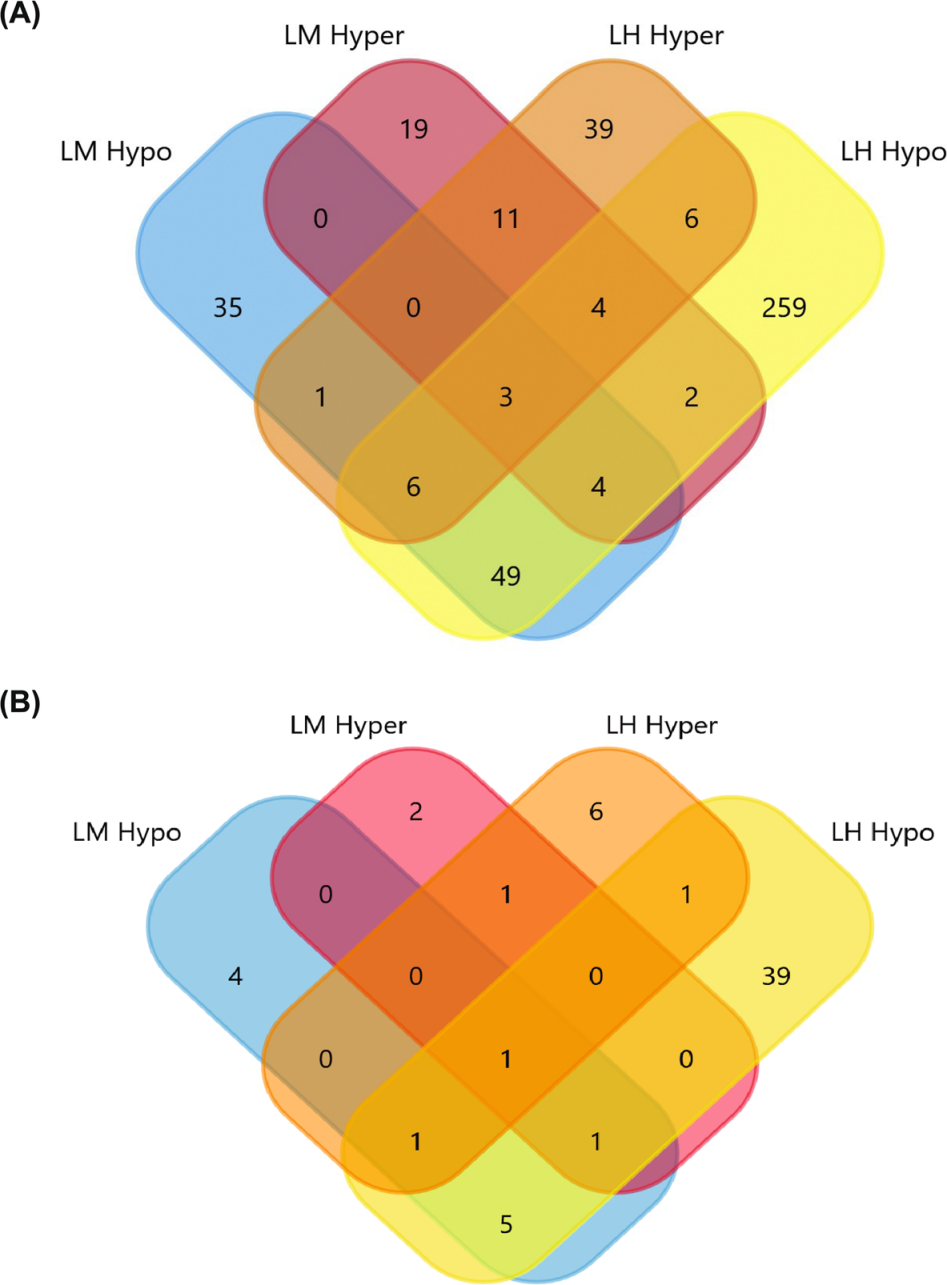
Annotation of the filtered DMCs to the closest TSSs and genes involved in embryonic organ development. A) Number of unique and in common closest TSSs (duplicate TSSs removed) to the filtered DMCs in the different groups. B) Number of unique and in common corresponding genes involved in embryonic organ development in the different groups. LM: low – medium, LH: low – high, Hyper: hypermethylated cytosines, Hypo: hypomethylated cytosines

Next, the nearest transcription start sites (TSS) to filtered DMCs and their corresponding gene information were extracted. This resulted in a greater number of TSSs in the hypo groups compared to the hyper groups, including 98, 43, 333 and 70 TSSs for LM hypo, LM hyper, LH hypo and LH hyper, respectively (Fig. 5a). Previous studies have indicated that although DNA damaged sperm cells could fertilize the oocyte; however, they could negatively affect the embryo development [10, 11]. Therefore, we were particularly interested in genes involved in embryonic organ development and functional annotation indicated that a greater number of these genes are observed in the LH comparison compared to the LM comparison. Interestingly, the majority of these genes were associated with the hypo groups (Fig. 5b).

### Pathway analysis

**Fig. 6:**
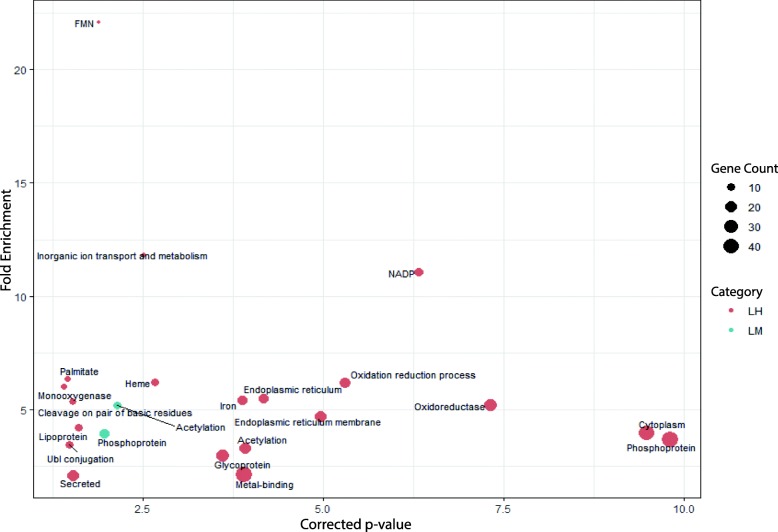
Pathway analysis for closest TSSs to filtered DMCs in hypo groups. GO terms are plotted in function of their corrected *p*-value (x-axis) and fold enrichment (y-axis). Gene count size key shows the number of genes identified for that particular GO term. LM: low – medium, LH: low – high, Hyper: hypermethylated cytosines, Hypo: hypomethylated cytosines, FMN: flavin mononucleotide, NADP: Nicotinamide adenine dinucleotide phosphate)

After adjusting the *p*-value for multiple testing in pathway analysis, genes with nearest TSSs to filtered DMCs in the LM hypo group were significantly associated with acetylation and phosphorylation pathways. A total number of 20 important biological process including acetylation, phosphorylation, membrane function, metabolic cascade and antioxidant defence system were connected to TSSs extracted from the LH hypo comparison (Fig. 6). However, none of the extracted GO terms exhibited significant association in the hyper groups. In the MH comparison, 61 and 148 TSSs were linked to hyper- and hypomethylated Cs, respectively, but, none of the identified TSSs were linked with any pathways (Additional file 3).

## Discussion

In the current study, various sperm DNA integrity parameters from liquid preserved boar semen samples with low, medium and high DFI values were analysed. Furthermore, sperm DNA methylation profiles were investigated using RRBS.

Our results indicate that of all investigated parameters, DFI, the most widely studied DNA integrity parameter, exhibited the greatest contrast between individuals, with higher levels at Day 4 compared to Day 0. However, it did not correlate well with protamine deficiency, which is in contrast to a previous study on bull sperm cells, where it was reported that DFI exhibited a significant and positive correlation with protamine deficiency [15]. In addition, the results showed that DFI has a significant but weak correlation with free thiols and disulphide bonds. Previously, it was shown that a slight reduction in disulphide bonds led to tighter DNA packaging in bull sperm cells, but a complete loss of disulphide bonds resulted in sperm DNA decondensation [16]. To our knowledge, the present study is the first showing that, disulphide bonds, total thiols and protamine levels are significantly decreased during liquid preservation of boar semen. Also, as there are multiple additional factors influencing DNA integrity such as abortive apoptosis and oxidative stress [17], the relationship between sperm DNA fragmentation and protamine or disulphide bonds should be further investigated using a larger number of samples.

The average percentage of bisulphite conversion rate was 99.8% (Table 1), which is higher than the previously reported results for similar libraries in pigs [13, 18] and slightly higher compared to results for bovine RRBS libraries [19]. Bisulphite treatment converts non-methylated Cs into Us and a higher conversion rate indicates more efficient and stable bisulphite conversion process. Incomplete conversion leads to counting of unmethylated Cs as methylated Cs, hence yielding false positive results. Therefore, it is recommended that the conversion rate must be over 99.5% and as close as possible to 100% [20]. Furthermore, a good average read coverage of >19x (Table 3) was obtained, as read coverages between 5x – 15x have been recommended as an acceptable window for analysis of bisulphite sequencing data [21].

Mapping efficiency against a reference genome is an important factor when analysing bisulphite sequencing data. Low mapping efficiency is a sign of misalignment, non-unique mapping, low quality reads, adapter contamination or other issues with sequencing. Although different studies have used different bioinformatic pipelines, a unique mapping efficiency of 35–45% is considered acceptable and has been reported in previous RRBS studies [19, 22, 23]. The average unique mapping efficiency rate of 58% reported here (Table 1) is higher than the previously published results for RRBS in pigs [14, 18] and is a good starting point for downstream analyses. It has been suggested that double restriction digestion might improve the coverage of RRBS libraries [14, 22]. The use of *Msp*I and *Taq* α1 enzymes in this study supported this view, as we achieved higher coverage and mapping efficiency compared to previous studies.

The average percentage of global methylation in a CpG context was 33% among the 18 different boar semen samples (Table 3). An average of 40–50% global methylation has been previously reported in different pig tissues using RRBS [13, 14] and 70–80% in boar sperm using luminometric methylation assay (LUMA) [5, 24]. These differences could be explained by the nature of the RRBS method, as DNA in sperm cells is more compact compared to other cell types and because RRBS only focuses on the less methylated CG-enriched regions (CpG islands), which is a small subset of that compact genome. A similar DNA hypomethylation pattern has been previously described in bull sperm cells [24, 25]. Clustering analysis and Pearson correlation based on CpG_10_ methylation level revealed no distinct clustering and a very high positive correlation between samples (Fig. 2). These findings indicate minimal variation between the pooled samples and suggest that globally, DFI-specific differences in methylation are low in boar sperm cells. Genetic diversities notably affect DNA methylation, and the inbreeding coefficients provided by Topigs Norsvin (data not shown) shows little or no genetic co-relation between the seven boars. However, semen samples from the same boar and belonging to the same DFI group displayed distinct methylation levels (Fig. 2a), indicating that the overall lack of clustering was not solely caused by genetic diversity among the boars.

The analysis using DMCs of CpG_10_ revealed that the number of hypomethylated Cs in the LH comparison compared with the LM comparison increased considerably (Fig. 3). Association between DFI and sperm DNA methylation is not consistent across the literature. For example, it has been shown that global DNA methylation in human sperm cells is not associated [26] or has either a negative [27] or a positive association [28] with DFI. Sperm DNA methylome is determined during spermatogenesis, therefore establishing the causal relationship between sperm methylation level and DFI is challenging. However, it has been shown that oxidative stress increases DNA fragmentation in sperm cells [29, 30] and could convert the methylated Cs to 5-hydroxymethyl Cs, which is a prerequisite for DNA demethylation [31]. Although we did not measure oxidative stress in our samples, a higher level of hypomethylation could be explained by higher levels of oxidative stress in samples with high DFI.

The regional analysis showed that over 90% of filtered DMCs were annotated to be present in intergenic regions (Fig. 4). Intergenic regions are located between genes and their function is not well identified. However, recent studies have revealed that these regions could be transcribed and thereby regulate the function of RNA polymerase [32, 33]. In accordance with the present results, previous studies using Berkshire pig placenta with different litter size and bull semen have also demonstrated that a greater percentage of DMCs, is present within the intergenic regions [24, 34]. Although the percentage of the filtered DMCs present in the intergenic regions is not different between LM and LH groups, further research should focus on these regions and their methylation signature.

In contrast to intergenic regions, the percentage of annotated filtered DMCs present in the promoter and gene body elements (exon and intron) showed clear group differences, as none of filtered DMCs were present in the promoter and exons in the LM comparison compared to the LH comparison (Fig. 4). It is well documented that DNA methylation in the promoters inhibits transcription and in the gene body promotes transcription initiation [35]. Therefore, these results suggest that DNA methylation is more likely to regulate genes in the LH group. However, as sperm cells are transcriptionally inactive, future research must focus on gene expression in embryos obtained via fertilizing the oocytes with high and low-level DFI sperm cells.

Sperm DNA integrity has been linked with several important biological functions in sperm cells and offspring. We have recently shown that litter size in both Norwegian Duroc and Landrace breeds is negatively correlated with DFI [7]. Fertilization and embryo development are complex biological phenomena and how sperm DNA fragmentation could affect the process is not well understood. However, using pathway analysis we identified some of the important genes and pathways, which might better explain why sperm cells with high DFI result in low field fertility. For instance, in comparison with fertilization, a larger number of genes involved in different organ developmental process were found in the LH comparison compared to the LM comparison (Fig. 5B). The number of annotated TSSs and identified pathways in the LH comparison was higher than the LM comparison and all pathways identified in the LM comparison were present in the LH group. Furthermore, some important genes involved in the development of central nervous system and in utero embryonic development were exclusively identified in the LH hypo group. In addition, several important pathways involved in membrane function, metabolic cascade and antioxidant defence system were identified (Fig. 6). Similar pathways have previously been identified using RNA-Seq data taken from testicles in boars with different levels of DFI [36] and using DNA methylation data obtained from Berkshire pig placenta with different litter sizes [34].

## Conclusion

Briefly, results in this study show how different DNA integrity parameters in boar sperm cells correlate with each other and to what extent DNA methylation is different in sperm cells with different levels of DFI. Our results demonstrate that DFI is only slightly correlated with other sperm DNA integrity parameters. Based on RRBS analysis, the number of DMCs, as well as the number of linked pathways in the LH group, were higher compared to the LM group. This suggests that samples with higher DFI could potentially have a greater range of affected biological pathways including those involved in embryo developmental processes.

## Materials and methods

In the current study, sperm phenotypic traits linked to DNA integrity including DFI, protamine deficiency, free thiols, total thiols, and disulphide bonds were analysed in boar semen both on the day of collection (Day 0) and upon 96–108 h storage (Day 4). As DFI exhibited the greatest contrast between samples compared to other phenotypic traits, samples selected and grouped based on their DFI level on Day 4, were subjected to RRBS.

### Sample collection

Ejaculates (*n* = 28) were collected from seven Norwegian Landrace (NL) boars (240–570 days old), using the “gloved hand” technique. The boars were housed and cared for according to international guidelines and regulations for keeping pigs in Norway, at Norsvin artificial insemination (AI) station, in Hamar, Norway. After semen collection, ejaculates with motility above 70% and morphological abnormalities below 20%, were further diluted to 28 × 10^6^ cells/ml using either the commercial extender Tri-X-cell® (IMV technologies, L Aigle, France) or Androstar® Plus extender (Minitube, Tiefenbach, Germany). According to Norsvin recommendations, semen doses can be stored at 18 °C up to 4 days (96–108 h), prior to AI and in Norway most of the semen are used at Day 3 and 4. Aliquots (1 ml) from both Day 0 and Day 4 semen samples were frozen at − 80 °C until analyses. In order to mimic the status on the day of use in the herds and to consider the worst-case scenario, only semen samples from Day 4 with high, medium and low DFI contrasts were included for RRBS analyses.

### Sperm phenotypic analyses

#### Sperm DNA fragmentation analysis

DFI was analysed using sperm chromatin structure assay (SCSA) according to Evenson et al. (2001) [37] and Boe-Hansen et al. (2005) [38] with minor modifications. In brief, snap-frozen semen samples were thawed in a 37 °C water bath and diluted to 2 × 10^6^ cells/ml using TNE buffer (10 mM Tris-HCl, 0.1 M NaCl, 1 mM EDTA, pH 7.4). This procedure was followed by denaturation of sperm DNA for 30 s by adding an acid solution (0.38 M NaCl, 80 mM HCl, 0.1% Triton-X 100, pH 1.2) and subsequently the samples were stained with acridine orange (AO) staining buffer (37 mM citric acid, 0.126 M Na_2_PO_4_, 1.1 μM EDTA, 0.15 M NaCl and 0.6 μg/ml of AO, pH 6.0). For each ejaculate, two technical replicates were analysed. Samples were protected from light and incubated at room temperature for 3 min prior to analysis of 5000 cells per technical replicate by a flow cytometer equipped with a blue laser (488 nm) (Cell Lab QuantaTM SC MPL flow cytometer, Beckman Coulter, Fullerton, CA). The flow cytometry instrument was AO-saturated prior to analysis, by running the AO equilibration solution (1.2 ml AO staining solution and 400 μl acid detergent solution) for 5 min. Green and red fluorescence signals were collected with a 525 nm band pass and a 670 nm long pass filter, respectively. To control laser stability, mean green and red fluorescence signals were re-set to 425 ± 5 and 125 ± 5, respectively, using a boar semen reference sample with known DFI at the start of the analysis and after every fifth sample. According to SCSA principle, after AO staining, double- and single-stranded DNA, emits green and red fluorescence, respectively. The percentage of red and green fluorescence was determined using the Cell Lab QuantaTM SC MPL analysis software (Beckman Coulter, Software version 1.0 A). Based on the ratio of red/(red + green), the DFI was calculated.

#### Protamine deficiency assay

The level of protamine deficiency in boar spermatozoa was assessed using Chromomycin A3 (CMA3; Sigma-Aldrich), according to the method described by Zubkova et al. (2005) [39] with minor modifications. Briefly, frozen-thawed samples (in duplicate) were diluted in TNE buffer (2 × 10^6^ cells/ml) and washed with PBS by centrifugation (300 x g; 10 min). The sperm pellet was resuspended in 100 μl McIlvaine’s buffer (17 ml 0.1 mol/l citric acid mixed with 83 ml 0.2 mol/l Na_2_HPO_4_ and 10 mmol/l MgCl_2_, pH 7.0) containing 0.25 mg/ml CMA3. Prepared samples were protected from light and incubated for 20 min at 37 °C. Stained sperm samples were washed in 500 μl PBS by centrifugation (300 x g; 10 min) before the pellets were resuspended in 500 μl PBS containing 4 μl propidium iodide (PI, 2.4 mM solution; Molecular Probes). All samples were analysed in a flow cytometer (FACSVerse, BD Biosciences) equipped with a blue laser (488 nm). The sperm cell population was gated using FSC and SSC and sperm cells were further identified by PI positive signal collected via 586/42 bandpass filter. The CMA3 fluorescence from gated cells was collected through a 528/45 bandpass filter after excitation with a violet laser (405 nm).

#### Free, total thiols and disulphide bonds status

Free and total thiols, as well as disulphide bonds in boar spermatozoa, were analysed using monobromobimane (mBBr; Molecular Probes), as described by Zubkova et al. (2005) [39] and Seligman et al. (1994) [40] with some modifications. Briefly, frozen-thawed spermatozoa (in duplicate) were diluted in TNE buffer (2 × 10^6^ cells/ml) and divided into two tubes, each containing 1 × 10^6^ cells. Tube 1 was loaded with 1 mM/l of 1,4-dithiothreitol (DTT, Sigma-Aldrich) and was incubated for 10 min at 37 °C while no DTT was added to tube 2. Both tubes were centrifuged for 10 min at 300 x g and the pellets were resuspended in 100 μl PBS containing 0.5 mM of mBBr solution. This procedure was followed by incubating both tubes for 10 min at 37 °C, protected from light. Stained spermatozoa were washed in 500 μl PBS and centrifuged for 10 min at 300 x g. The pellets were resuspended in 500 μl PBS and analysed with a FACSVerse flow cytometer (BDBiosciences) as described above.

In order to calculate disulphide bonds, free thiols fluorescence signals (mBBr fluorescence from non-DTT treated samples) were subtracted from total thiols fluorescence signals (taken from the corresponding DTT treated samples), then the value was divided by two.

### RRBS library preparation and sequencing

RRBS library preparation and sequencing were performed according to Boyle et al. (2012) [41], with slight modifications and consisted of the following steps.

#### Genomic DNA extraction

DNA from Day 4 frozen semen samples was isolated using Maxwell 16 Benchtop DNA extraction instrument (Promega Corporation, USA). Isolated DNA was quantified using Qubit dsDNA BR assay kit (Thermo Fisher Scientific, USA) and further diluted to 20 ng/μl in low TE media [10 mM Tris, pH 8.0 (Calbiochem, USA), 0.1 mM EDTA, pH 8.0 (Calbiochem, USA)] at Biobank AS, Hamar.

#### Pre-treatment of genomic DNA

Genomic DNA (400 ng) was digested overnight at 37 °C using *Msp*I and *Taq* α1 enzymes (New England Biolabs, USA). Gap filling and A-tailing were conducted by adding 1 μl of Klenow fragment (New England Biolabs, USA) as well as 1 μl of 10 mM dNTP mixture (New England Biolabs, USA) into each digestion reaction well in a reaction plate. The samples were incubated (30 °C for 20 min, 37 °C for 20 min, 10 °C indefinitely) in a thermocycler without the heated lid and at the end, 300–500 bp fragments were further selected using SPRI AMPure XP beads (Beckman Coulter, USA). Thereafter, 2 μl of NEXTflex™ Bisulphite-Seq barcodes (Bio Scientific Corporation, USA) was added into each well and ligation reaction was performed by incubating the adapter ligase mixtures at 16 °C overnight. Prior to bisulphite treatments, fragments were further size selected by adding 60 μl (2x) 20% PEG 8000/2.5 M NaCl (Amresco Inc., USA) followed by incubation for 30 min at room temperature and the fragments were suspended in elucidation buffer. In order to evaluate the efficiency of adapter ligation as well as to determine the optimal PCR cycle number for later amplification, size-selected fragments were subjected to PCR amplification (using 10, 13, 16 and 19 cycles) with primers designed to bind with flank adapters. The optimal PCR cycles were found to be 13 and the PCR products were run on a 4–20% Criterion precast polyacrylamide TBE gel (Thermo Fisher Scientific, USA) and DNA bands were visualized by adding SybrGold (Thermo Fisher Scientific, USA) dye.

#### Sodium bisulphite modification procedure

Size-selected fragments (20 μl) were subjected to bisulphite conversion using the EpiTect kit (QIAGEN, Germany) following the manufacturer’s protocol designated for DNA extracted from FFPE tissues.

#### Post-bisulphite conversion procedure

Bisulphite-converted DNA was cleaned up according to the protocol described in the QIAGEN EpiTect kit and [42]. Cleaned up and converted DNA was further amplified using 13–16 cycles of PCR. At the end, library concentration was quantified using the PicoGreen dsDNA absorbance method.

#### Illumina sequencing

Elucidated samples were sequenced using the Illumina HiSeq 2500 in the single end (1 × 100 bp) mode.

### Bioinformatic analysis

#### Quality control and adapter trimming

Illumina adapter sequences and low-quality bases (below 20 bp and Phred score of 20) were trimmed from raw single end Illumina reads using Trim Galore! software (v 0.4.4) [43].

#### Clean reads alignment

In this study, the recently published *Sus scrofa* 11.1 genome [44] was used as reference genome. in silico analysis using CLC Genomics Workbench showed that the new pig *Msp*I digested RRBS genome was 3% of the total genome, which is an increase of 16% compared to the previous version [14]. Clean and high-quality reads were mapped to the reference genome using default parameters (−n 0 -l 20 and --score-min (L, 0, − 0.2)) with the Bismark tool and using bowtie2 aligner (v 0.19.0) [45]. Bismark provided the global CpG methylation level for each library by calculating the methylation of each covered Cs using following formula: % methylation = 100 * number of methylated Cs / number of methylated Cs + number of unmethylated Cs.

#### Methylation analysis

SAM sorted alignment files from Bismark were analysed using the methylKit package (v 1.6.1) [46] in Rstudio (v 1.1.453) for Linux. Based on descriptive statistics such as CpG coverage and percent methylation, reads containing CpGs with more than 99.9th percentile coverage were filtered out and only reads with CpGs ≥10x coverage depth (CpG_10_) were considered for downstream analysis. Sperm samples with different DFI levels were divided into low (L), medium (M) and high (H) DFI based on our previous investigation [7]. For downstream analysis, samples from low and medium/high DFI groups were considered as control and test, respectively. The DMCs were identified using CpG_10_ between low – medium (LM), low – high (LH) and medium – high (MH) groups. Six samples each from L, H, and M were allocated to every group. The “min.per.group” option was set to four in unite function [46]. Furthermore, in order to determine DMCs with methylation difference > 25% and q-value < 0.01 (i.e., filtered DMCs), logistic regression analysis with a sliding linear model to correct for multiple comparisons was employed within the methylKit package. In this study, hypermethylation and hypomethylation are defined as differential methylation > 25% or < − 25% in the test group compared to the control group, respectively. In this study different methylation difference cut-offs were applied. In addition, using tiling function in methylKit, differentially methylated regions (DMRs) were identified (Additional file 4).

#### Annotation of DMCs

BED files for Gene and CpG annotation for *S. scrofa* 11.1 assembly were downloaded from the UCSC table browser [47]. The DMCs were further annotated using Genomation package (v 1.14.0) in Rstudio with TSS, nearest gene name, genes elements (exons, introns, promoter, intergenic regions) and CpG features (CGI, CpG shore, other). Promoters and CpG shore were defined as ±1000 bp and ± 2000 bp of the TSS and CGI, respectively.

#### Pathway analysis

In order to perform Gene Ontology (GO) analysis, extracted nearest TSS and gene ids to filtered DMCs, were submitted to DAVID Bioinformatics resources for functional annotation [48]. Fisher’s exact test was used to calculate gene enrichment. *p-*value was Benjamini corrected for multiple testing and was set to 0.05.

### Statistical analysis

Statistical analysis was performed in Rstudio, v 1.1.383 for windows. Linear mixed models within the lme4 package were established using phenotypic traits of sperm cells and preservation time as dependent and independent variables, respectively. In addition, individuals, animal age and season for the collection of semen as well as number of ejaculates were included as random effects. In order to minimize type I error, *p-*values were Bonferroni adjusted to 0.016 for total and free thiol as well as disulphide bonds. *p*-value for protamine deficiency and DFI was set to 0.05. Correlation between DFI, thiol profile and protamine deficiency as well as DFI and global CpG level was obtained via multiple linear regression model within the lme4 package and *p*-value was set to 0.05.

Results were plotted using GraphPad Prism (v 6.01 for Windows, GraphPad Software, San Diego, CA, USA). Volcano plots and Venn diagrams were constructed using Instant Clue (v 0.5.2 for Windows) and FunRich (v 3.1.3 for Windows) software, respectively [49, 50]. Pathway analysis results were plotted using ggplot2 package (v 3.1.0) in Rstudio [51].

## Supplementary information

**Additional file 1.** CpG basic statistics for all samples. Figure 1: CpG site coverage histogram, Fig. 2: CpG methylation distribution.

**Additional file 2.** Differential methylation and downstream analyses for Medium-High (MH) group. Figure 1: total DMCs and filtered DMCs for MH group, Fig. 2: annotation of filtered DMCs in MH comparison with genomic and CpG features.

**Additional file 3.** List of closest TSS to filtered DMCs and pathway analysis results in Medium-High (MH) group.

**Additional file 4.** Number of DMCs and DMRs after applying different methylation difference cut-offs.

## Data Availability

The datasets generated and analysed during the current study are available in the European Nucleotide Archive (ENA) repository, under project accession number PRJEB35306.

## Abbreviation

AI: Artificial insemination
AO: Acridine orange
CGI: CpG island
CMA3: Chromomycin A3
DFI: DNA fragmentation index
DMC: Differentially methylated cytosine
DMR: Differentially methylated region
DTT: Dithiothreitol
LH: Low - high
LM: Low - medium
LME: Linear mixed model
mBBr: Monobromobimane
MH: Medium - high
RRBS: Reduced representative disulphide sequencing
SCSA: Sperm chromatin structure assay
TSS: Transcription start site
