# Supplementary material for: DNA methylation patterns vary in boar sperm cells with different levels of DNA fragmentation
Source: BMC Genomics. 2019 Nov 27;20:897. doi: 10.1186/s12864-019-6307-8 (PMC6880426; doi:10.1186/s12864-019-6307-8)
Supplement: Supplementary file 1 — Additional file 1. CpG basic statistics for all samples. Figure 1: CpG site coverage histogram, Fig. 2: CpG methylation distribution. [file 12864_2019_6307_MOESM1_ESM.docx]

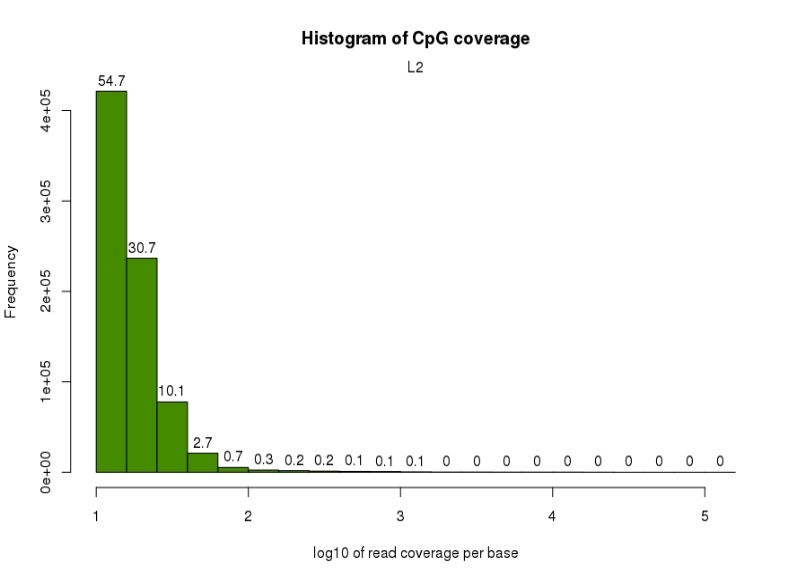

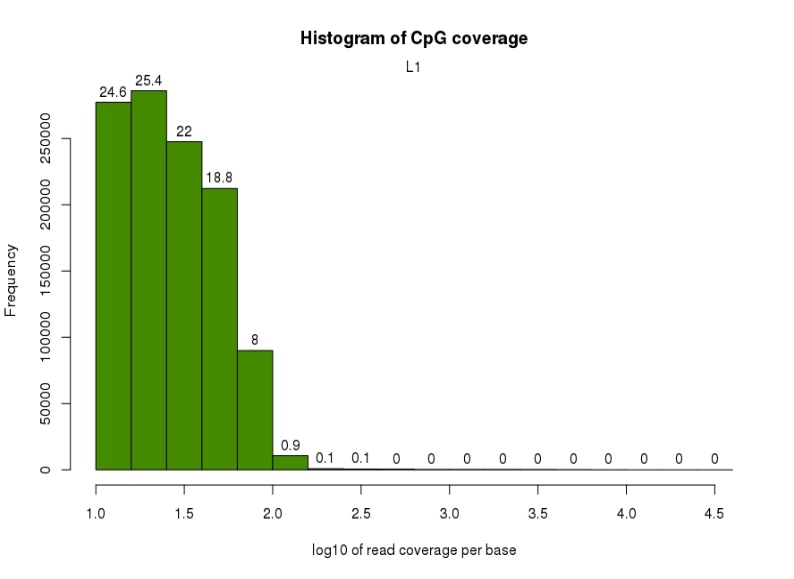

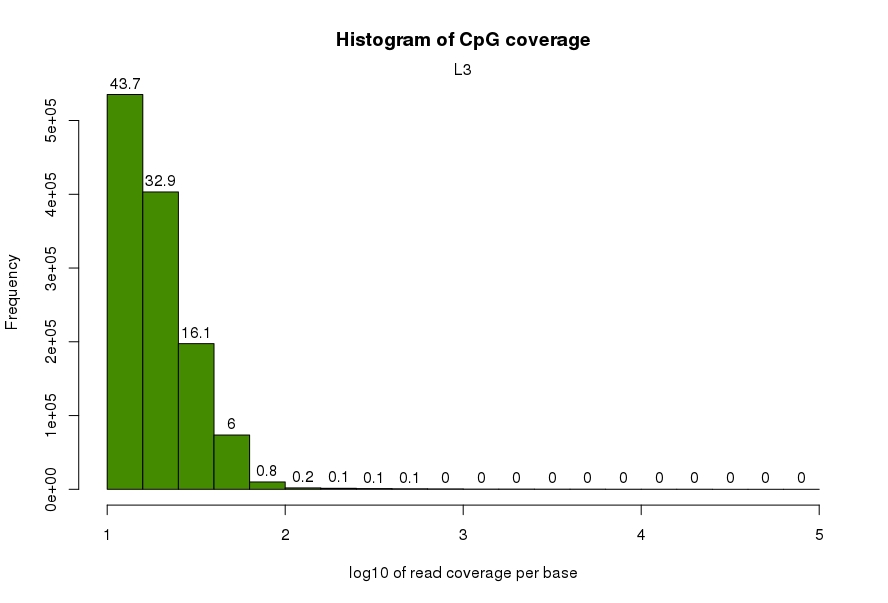

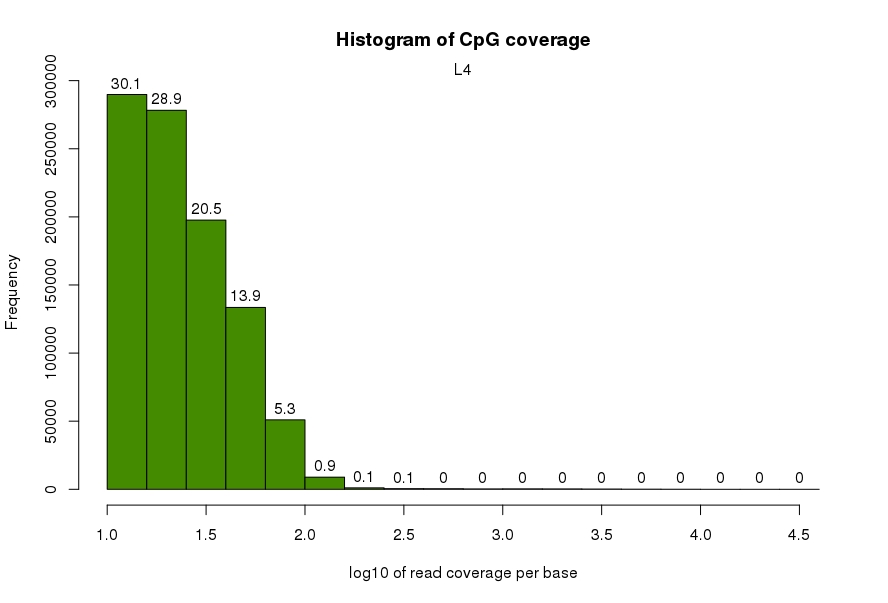

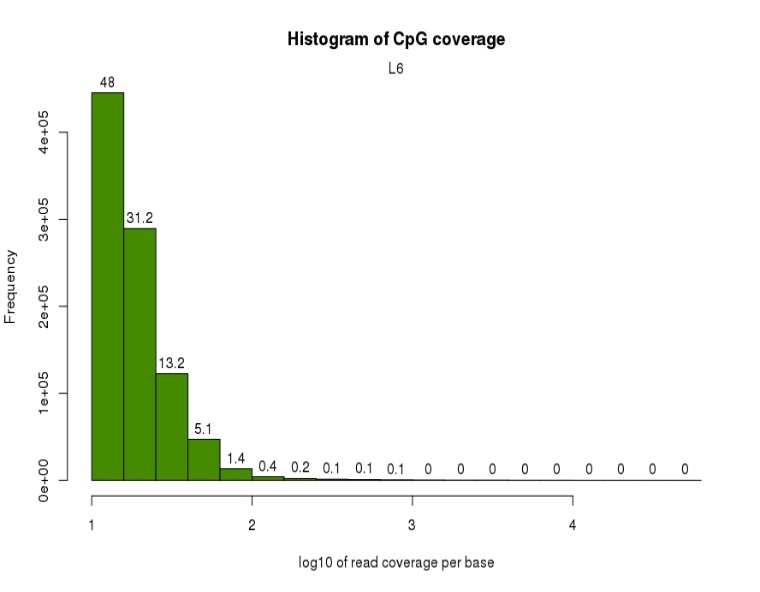

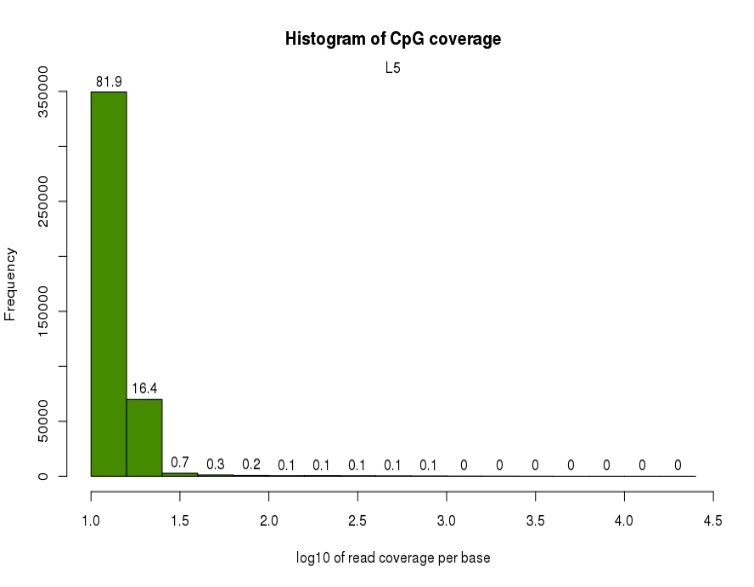


**Fig. 1**


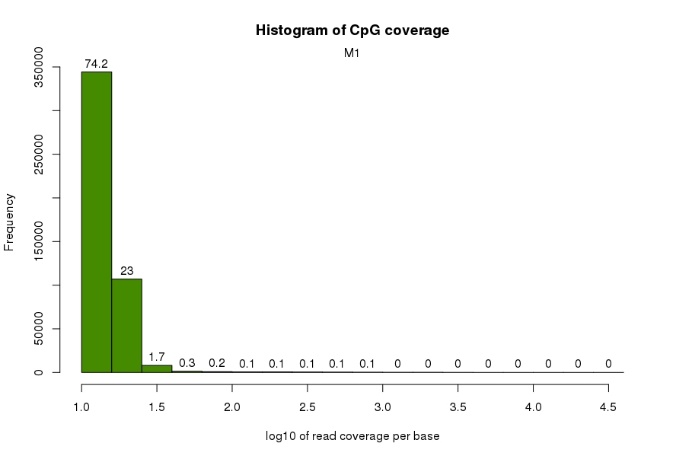

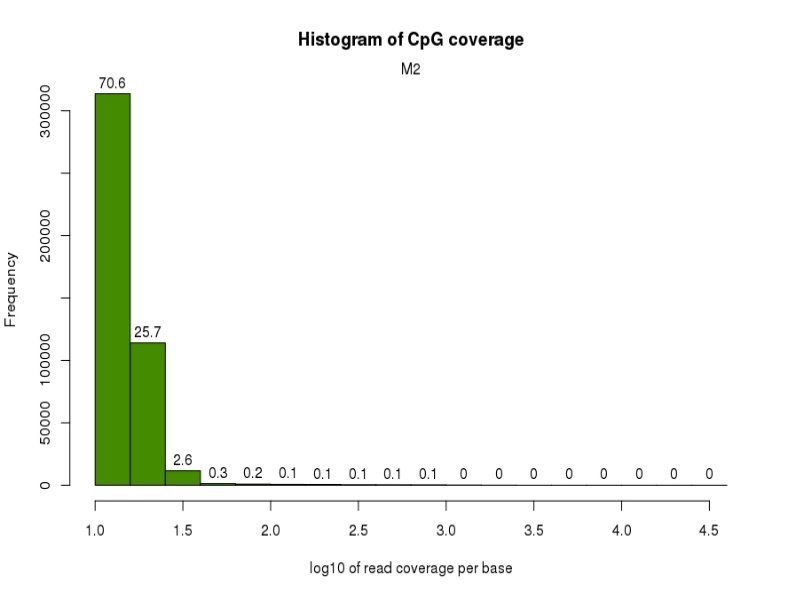

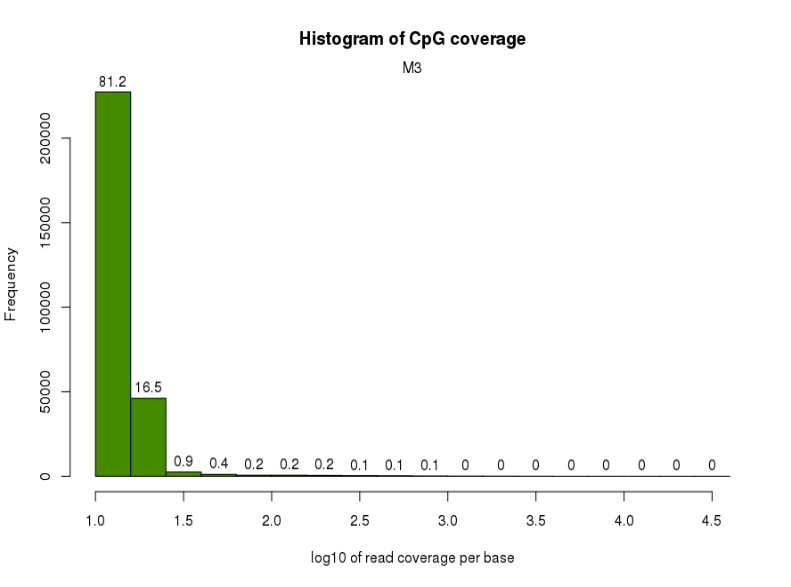

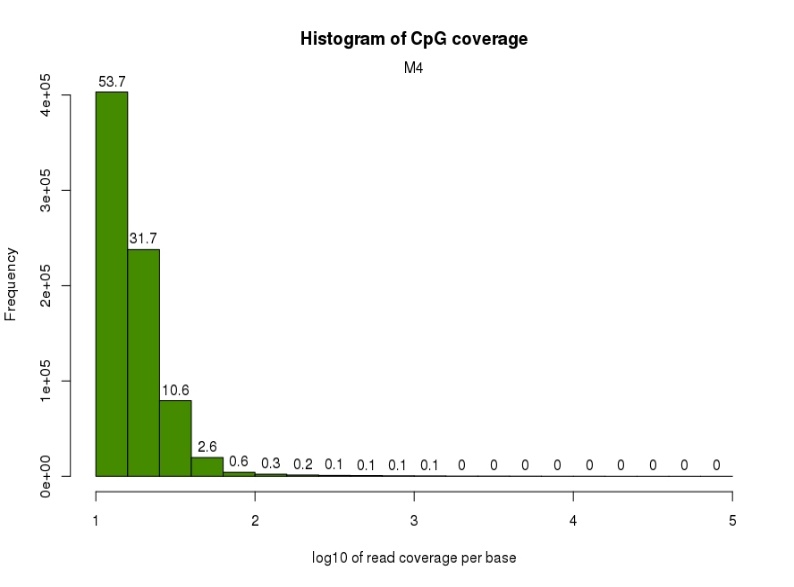

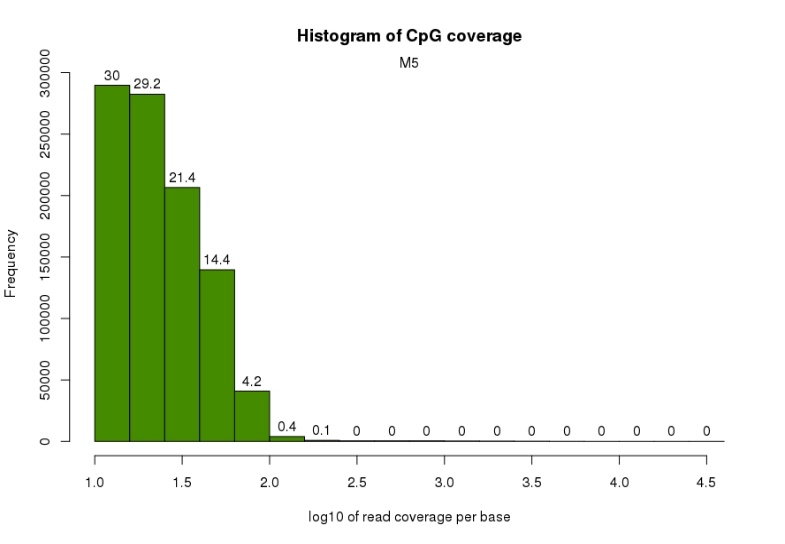

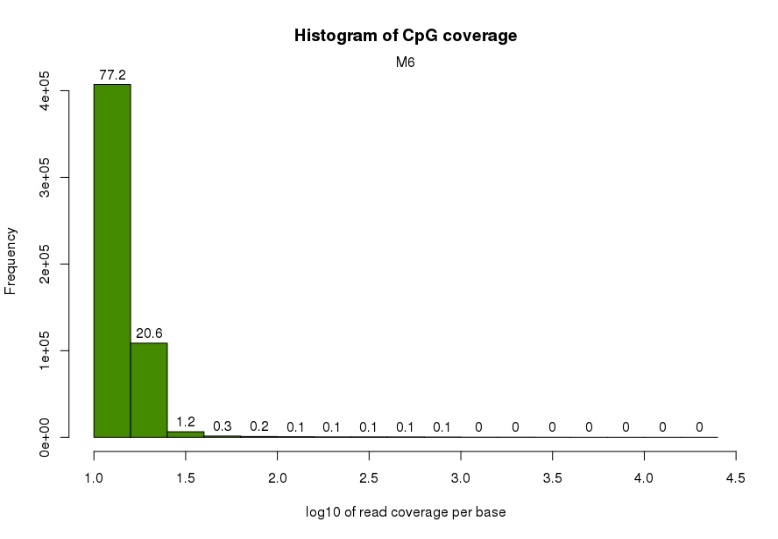

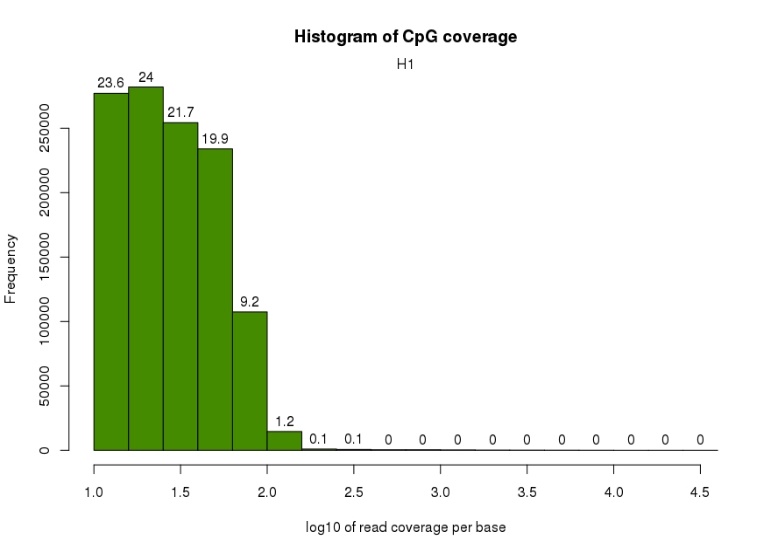

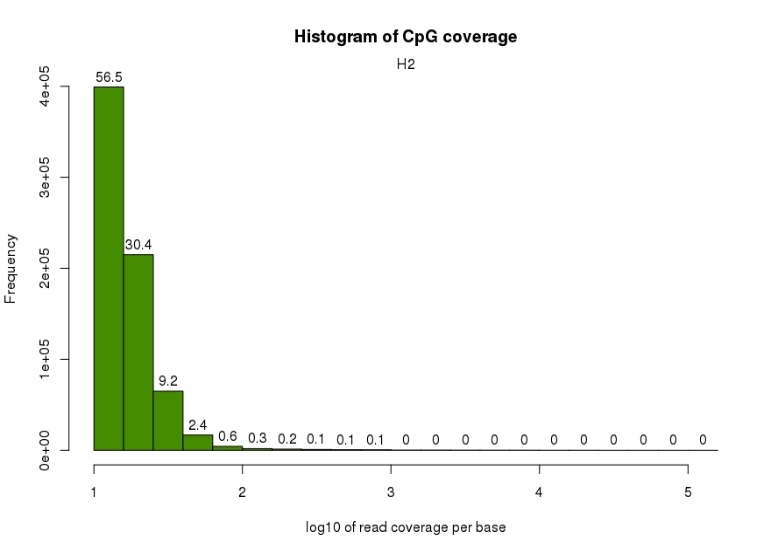

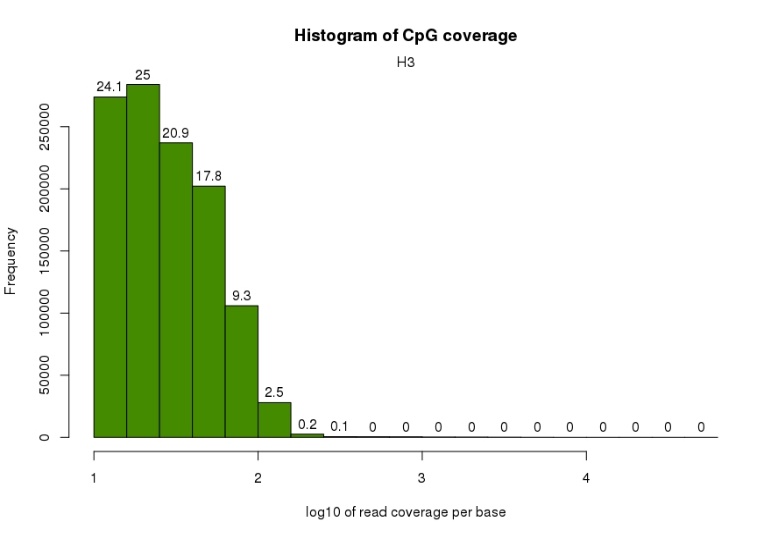

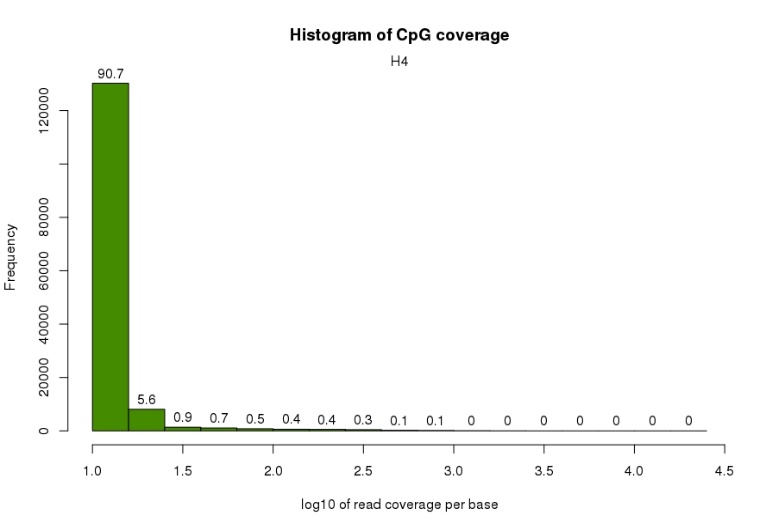

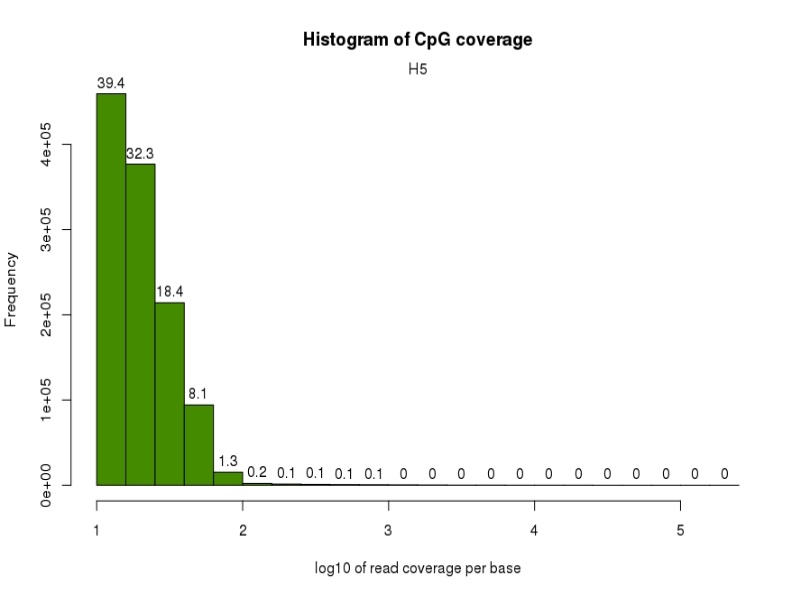

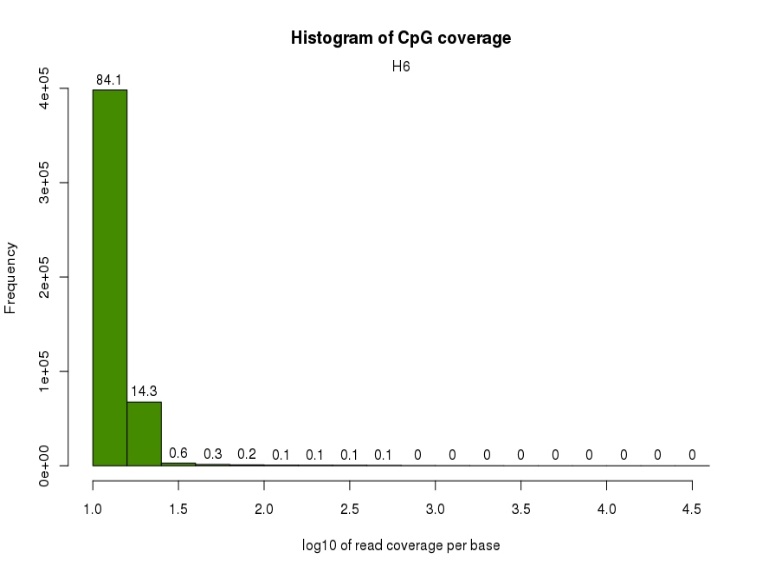


***Figure 1****.* ***CpG site coverage histogram in boar sperm cells****. The X-axis indicates log10 values corresponding to the number of reads per CpG. The numbers on the bars indicate the percentage in each respective bin.*

**Fig. 2**


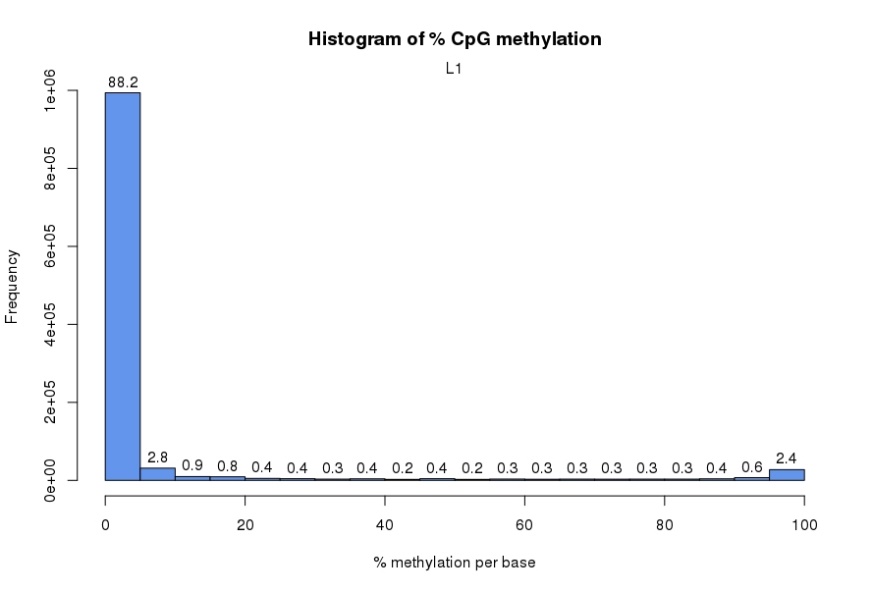

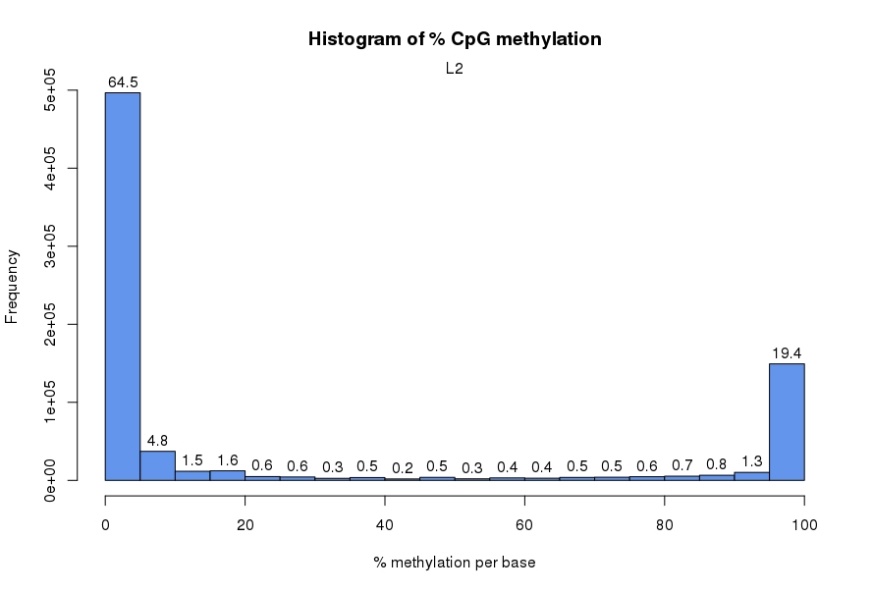

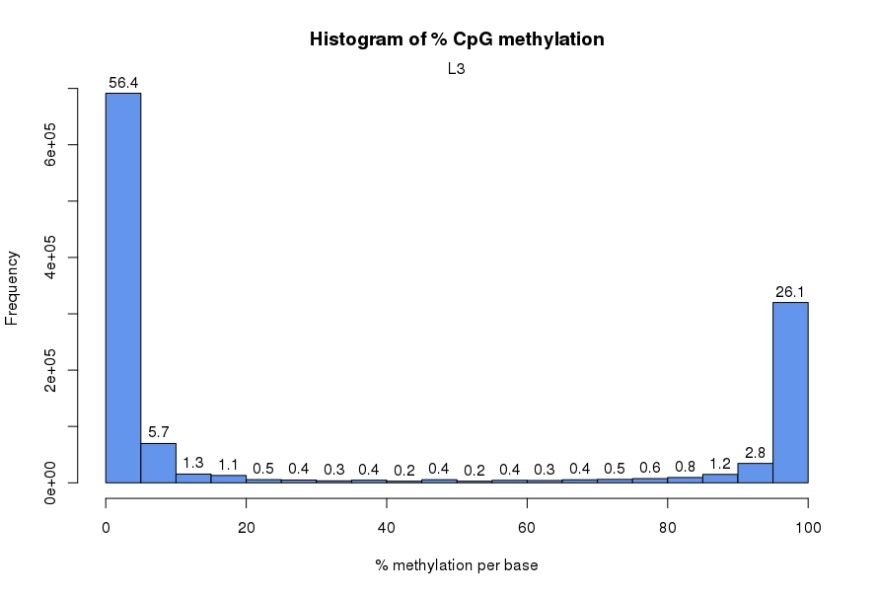

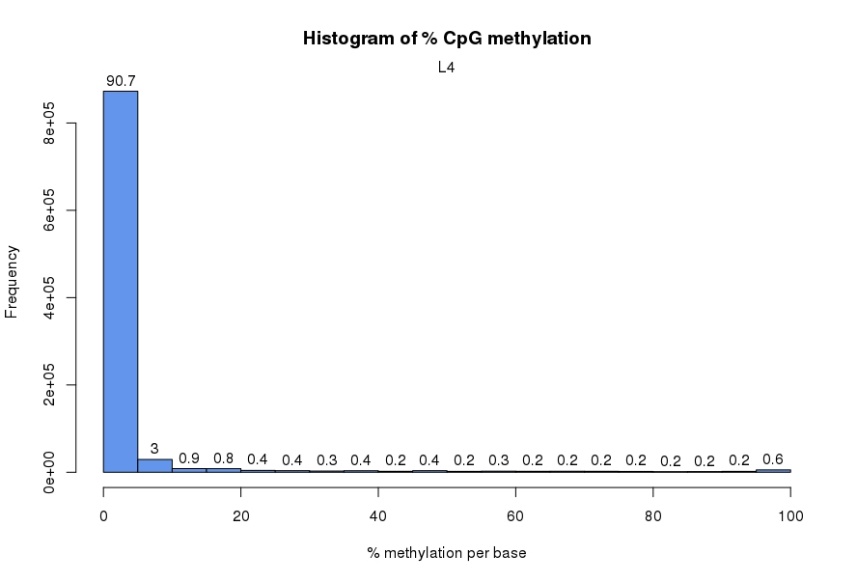

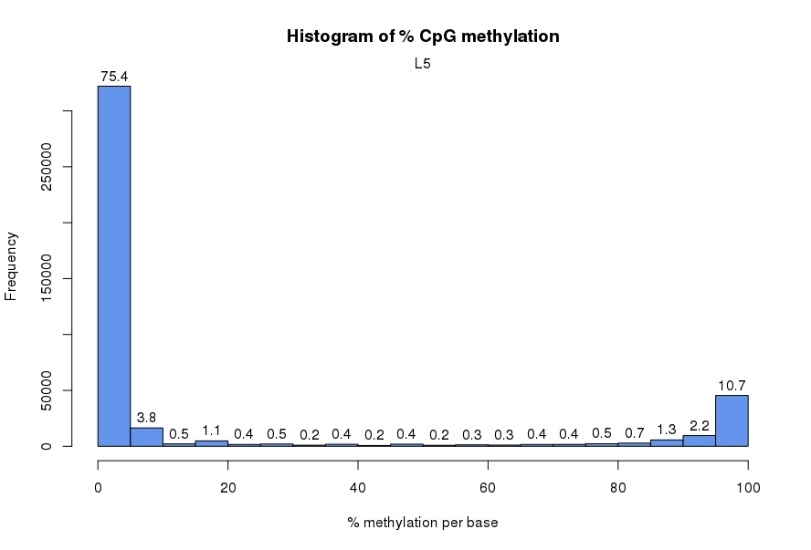

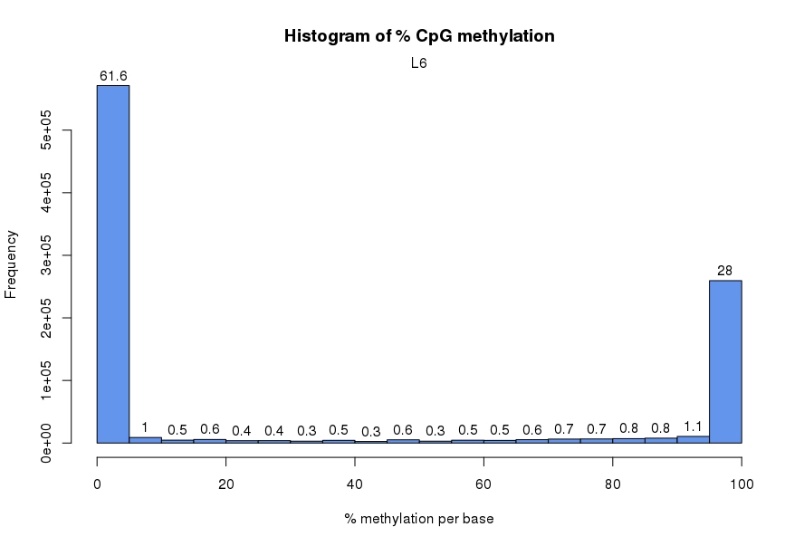

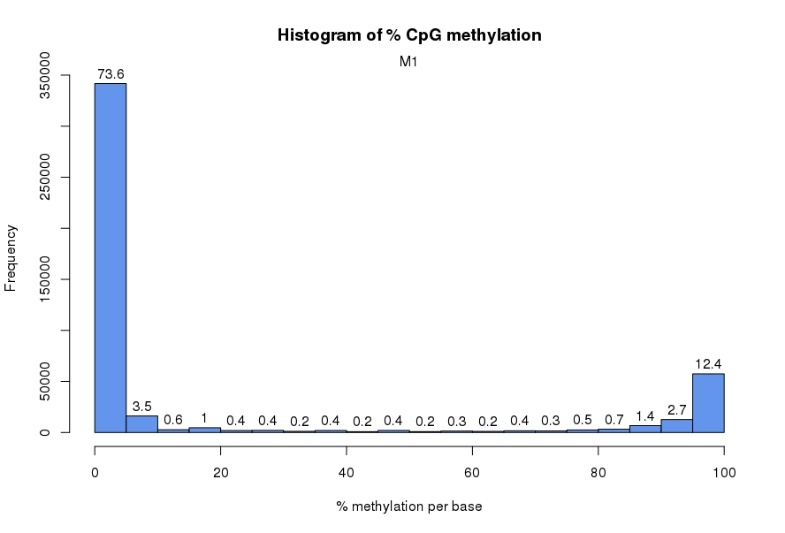

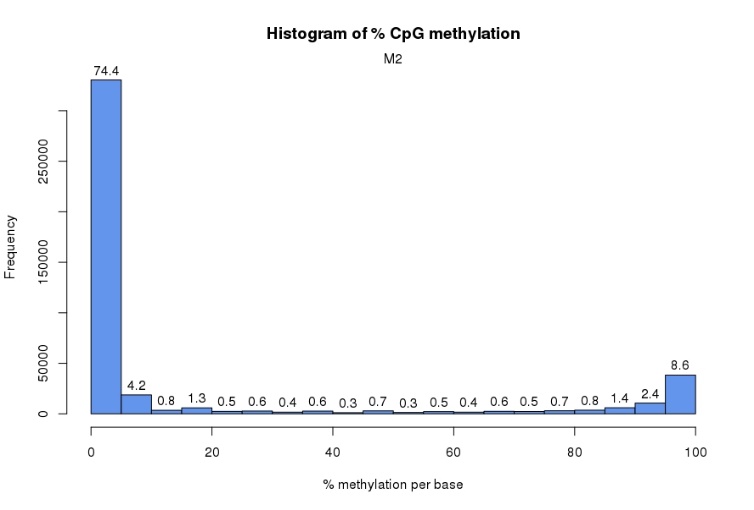

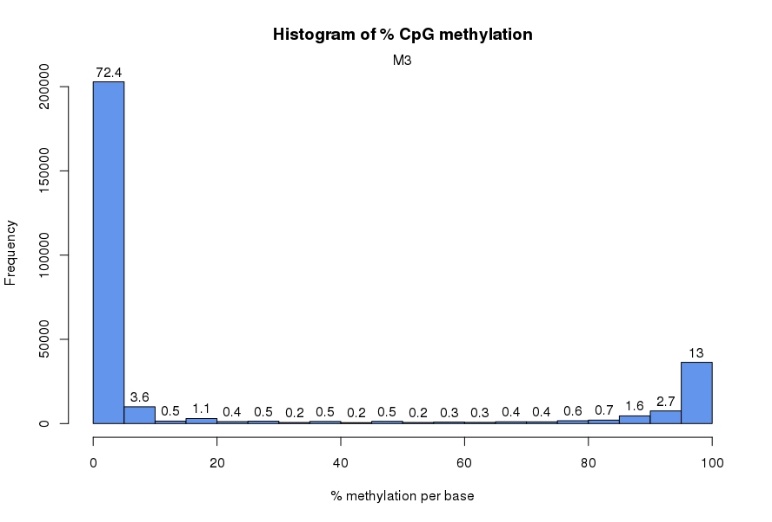

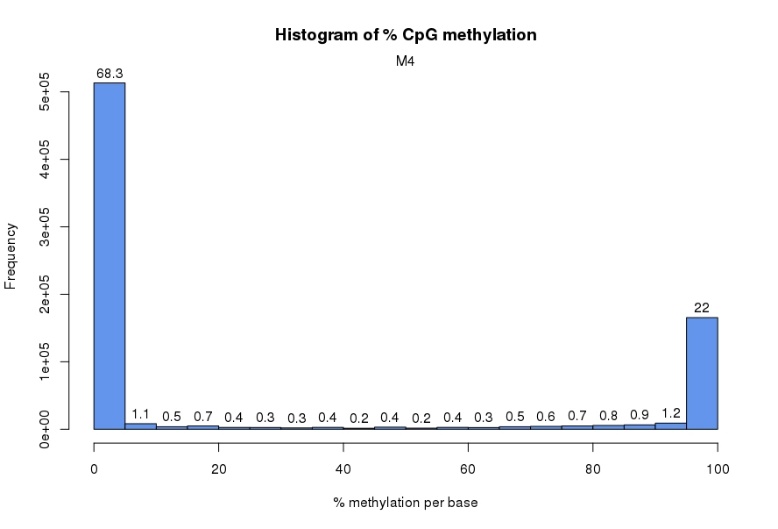

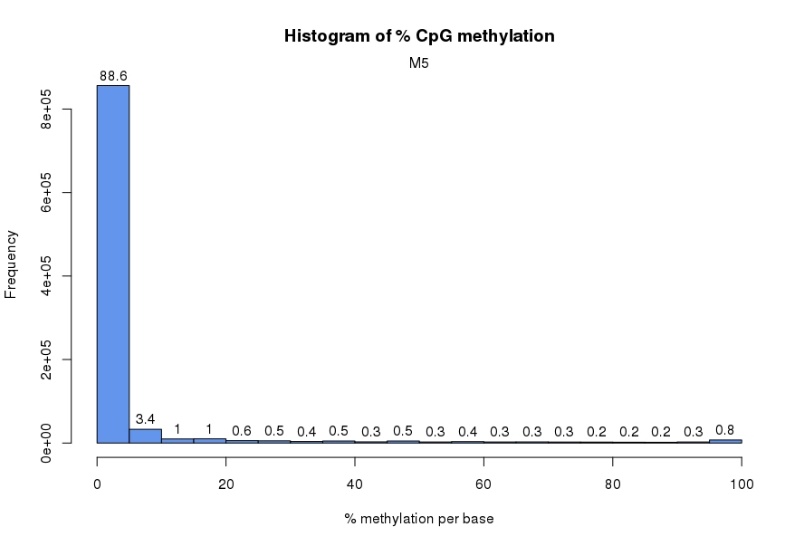

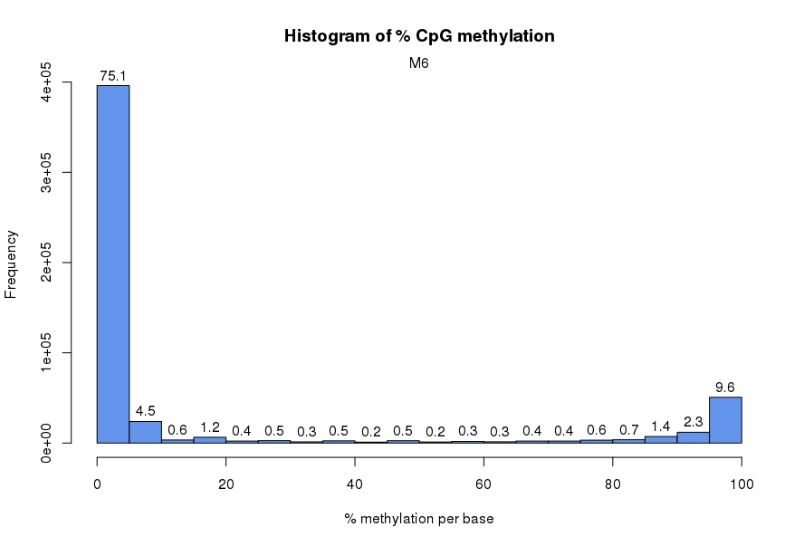

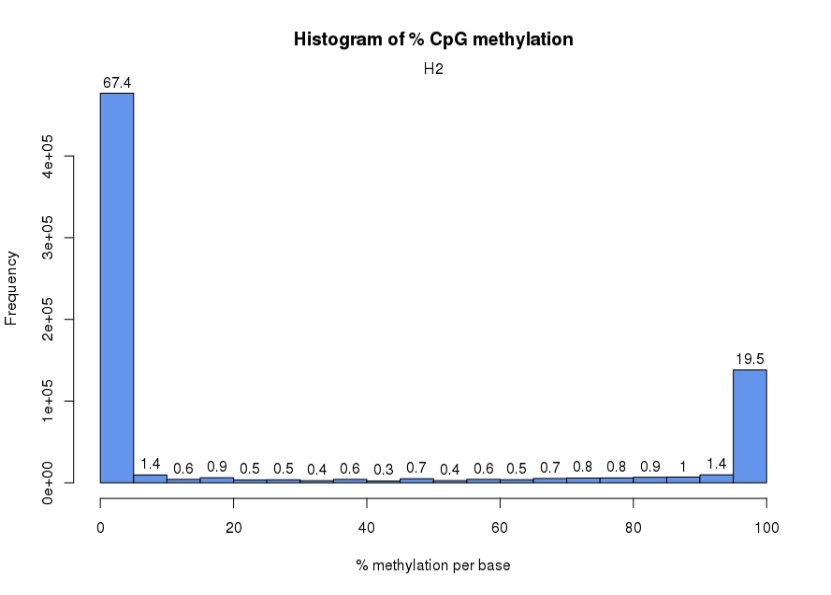

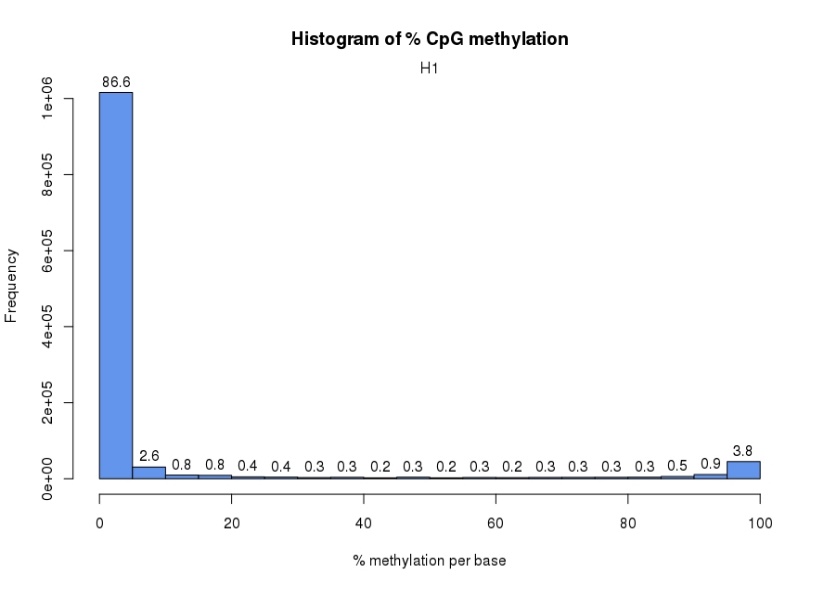

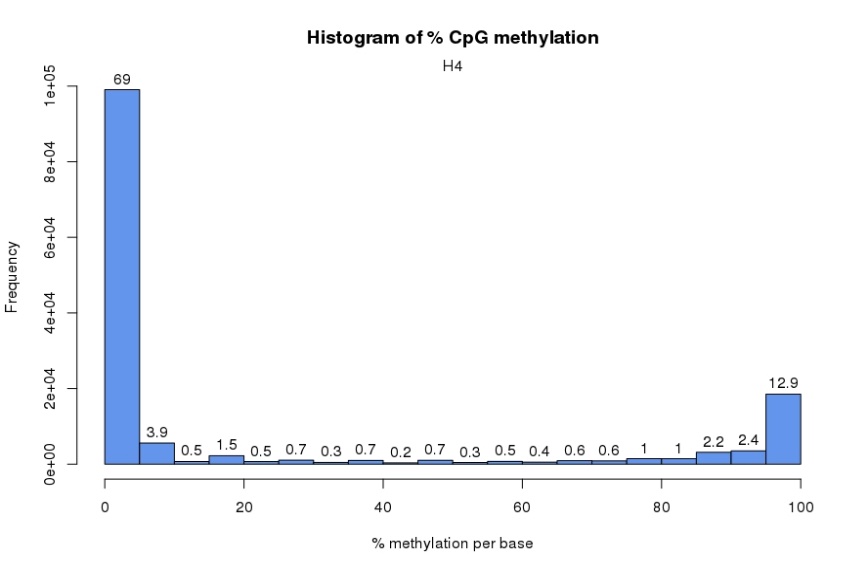

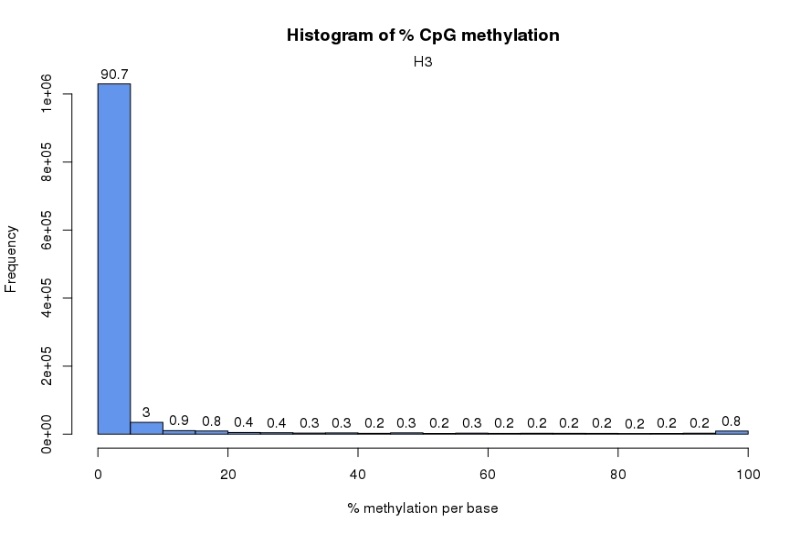

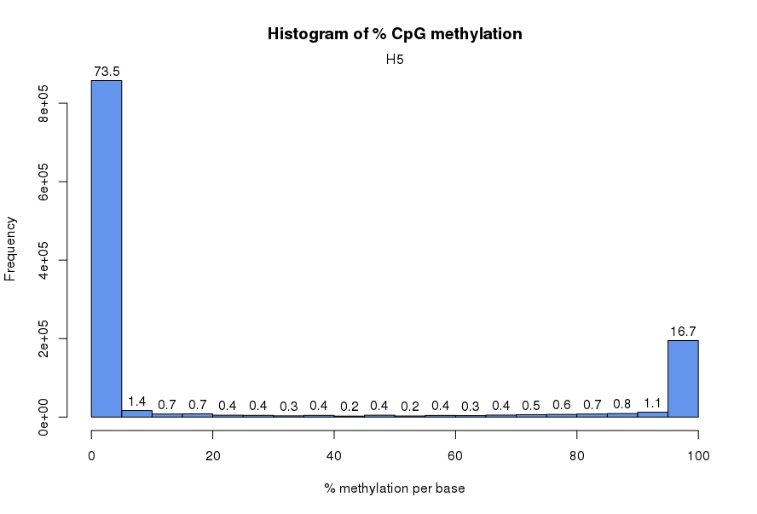

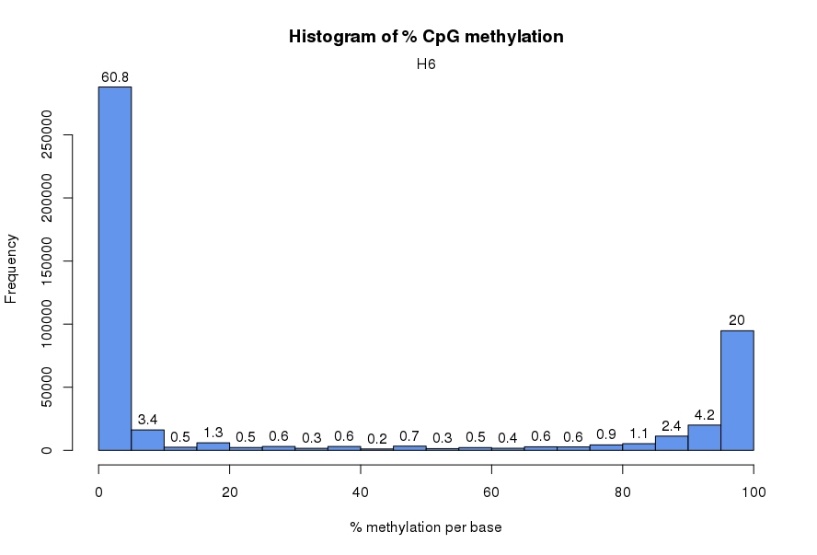


***Figure 2****.* ***CpG methylation distribution in boar sperm cells****. The X-axis indicates percent methylation for each CpG. The numbers on the bars indicate the percentage in each respective bin.*
