# Supplementary material for: DNA methylation patterns vary in boar sperm cells with different levels of DNA fragmentation
Source: BMC Genomics. 2019 Nov 27;20:897. doi: 10.1186/s12864-019-6307-8 (PMC6880426; doi:10.1186/s12864-019-6307-8)
Supplement: Supplementary file 2 — Additional file 2. Differential methylation and downstream analyses for Medium-High (MH) group. Figure 1: total DMCs and filtered DMCs for MH group, Fig. 2: annotation of filtered DMCs in MH comparison with genomic and CpG features. [file 12864_2019_6307_MOESM2_ESM.docx]

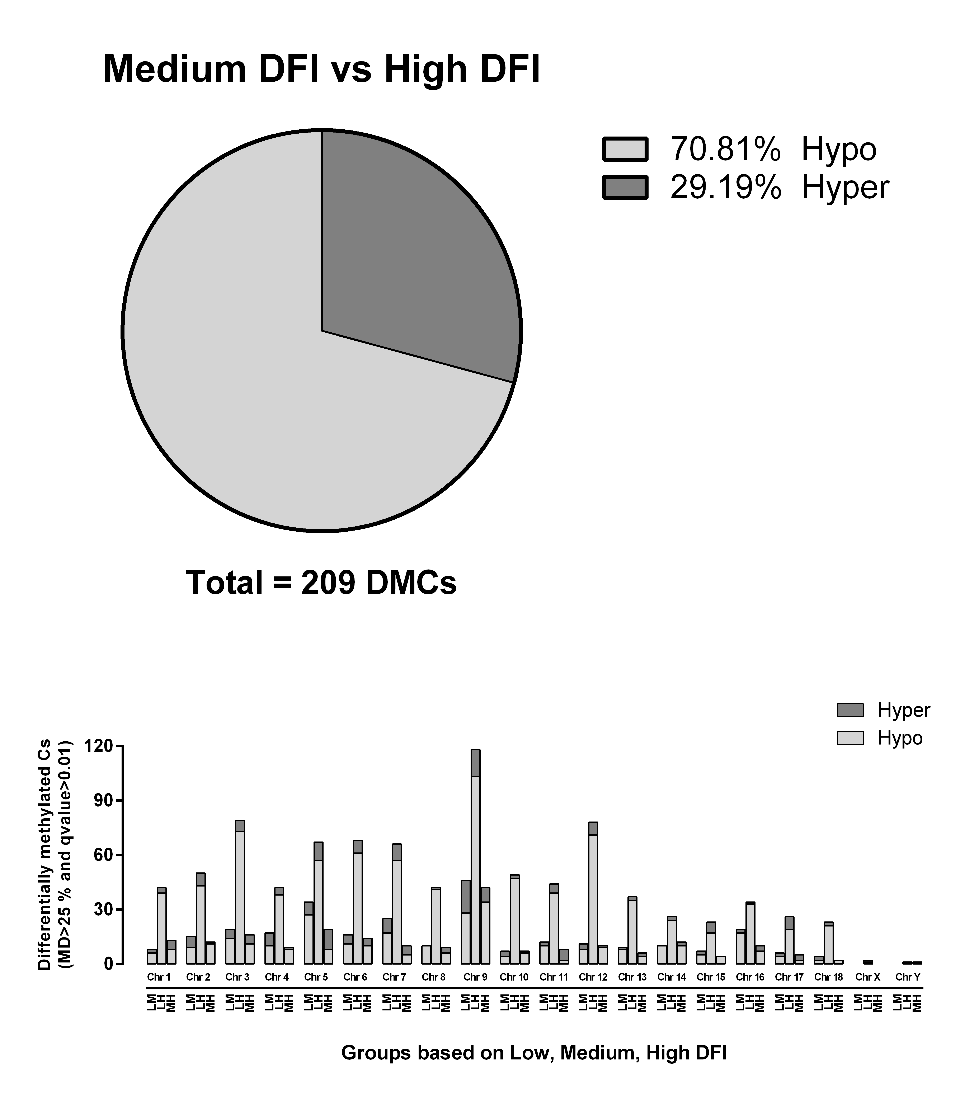

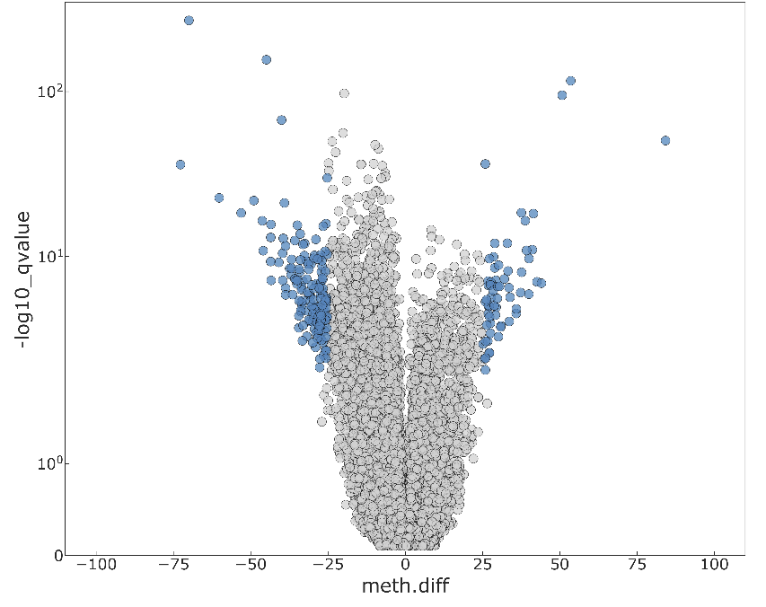


***Figure 1****.* ***Differential methylation analysis for sperm RRBS-based methylome for medium and high DFI group*.** *Volcano plot of methylation differences and p-values for all methylated cystosines (each circle). Blue circles represent DMCs with over 25% methylation difference and qvalue < 0.01 (filtered DMCs). Pie chart for filtered DMCs indicates higher percentage of hypomethylated cytosines in MH group.*

***Figure 2****.* ***Distribution of filtered DMCs obtained from MH group among gene and CpG features.***
