# Supplementary material for: DNA methylation patterns vary in boar sperm cells with different levels of DNA fragmentation
Source: BMC Genomics. 2019 Nov 27;20:897. doi: 10.1186/s12864-019-6307-8 (PMC6880426; doi:10.1186/s12864-019-6307-8)
Supplement: Supplementary file 4 — Additional file 4. Number of DMCs and DMRs after applying different methylation difference cut-offs. [file 12864_2019_6307_MOESM4_ESM.docx]

**Additional file 4**. We have also applied 10% and 25% cut-off levels while having min per group = 6. In addition using tile function, we applied 300bp window size, 300bp sliding window size to determine differentially methylated regions (DMR). Only regions with at least 4 Cs were included in the analysis.

In brief, regardless of the type criteria used, the main conclusions remained the same, which are:

- Majority of DMCs or DMRs are hypomethylated.
- By increasing the DFI both the total number and the number of hypomethylated DMCs/ DMRs increases.

|  | min per group | Methylation cut off | Number of DMCs/DMRs  Hypo | | Number of DMCs/DMRs  Hyper | |
| --- | --- | --- | --- | --- | --- | --- |
|  |  |  | LM | LH | LM | LH |
| DMCs | 4  (presented in MS) | 25 | 200 | 820 | 75 | 97 |
|  | 6 | 25 | 18 | 17 | 7 | 4 |
|  |  | 10 | 176 | 236 | 92 | 37 |
| DMRs  (300bp window,  min 4 mutual Cs / window) | 6 | 25 | 2 | 2 | 0 | 0 |
|  |  | 10 | 25 | 37 | 8 | 1 |
